# DRD1 and DRD2 dopamine-sensitive neurons in the central amygdala respond differently to rewarding and aversive stimuli

**DOI:** 10.1038/s41398-026-04228-7

**Published:** 2026-07-03

**Authors:** Łukasz Bijoch, Justyna Wiśniewska, Paweł Szczypkowski, Monika Pawłowska, Karolina Hajdukiewicz, Radek Lapkiewicz, Anna Beroun

**Affiliations:** 1 Nencki-EMBL Center of Excellence for Neural Plasticity and Brain Disorders: BRAINCITY, Laboratory of Neuronal Plasticity, Nencki Institute of Experimental Biology of the Polish Academy of Sciences, Warsaw, Poland; 2 Faculty of Physics, University of Warsaw, Warsaw, Poland; 3 Nencki-EMBL Center of Excellence for Neural Plasticity and Brain Disorders: BRAINCITY, Laboratory of Neurobiology, Nencki Institute of Experimental Biology of the Polish Academy of Sciences, Warsaw, Poland

**Keywords:** Long-term memory, Learning and memory

## Abstract

The ability to differentiate between rewarding and aversive stimuli is crucial for survival, yet the underlying neural mechanisms allowing animals to make such a distinction remain elusive. Here, using in vivo two-photon calcium imaging, we uncovered how the activity of dopamine-sensitive neurons in the medial nucleus of the central amygdala (CeM) is associated with appetitive and aversive experiences. We found that cocaine and sucrose strongly activated DRD1(+) neurons while suppressing DRD2(+) activity, whereas the aversive quinine stimulus predominantly engaged DRD2(+) neurons, particularly those not previously recruited by sucrose. Our findings suggest that DRD1(+) and DRD2(+) neurons differentially contribute to the processing of appetitive and aversive stimuli. Furthermore, by simultaneously monitoring facial expressions, we identified stimulus-specific behavioral responses to sucrose, quinine, and cocaine.

## Introduction

Dopamine is a neurotransmitter released in response to novel stimuli, both rewarding and aversive [1]. It is believed to play a crucial role in the initial stages of various types of learning that have emotional significance [2]. Additionally, dopamine is implicated in the addictive properties of drugs of abuse, as all such drugs affect its balance [3]. For example, cocaine blocks dopamine reuptake, causing it to remain in the synaptic cleft for a longer period, thereby prolonging its action on dopamine receptors [4].

Two primary dopamine receptor families have been identified: D1-type and D2-type, which exert opposing effects on neuronal excitability as D1(including Dopamine Receptor D1 and D5, DRD1 and DRD5) receptors promote neuronal activation, whereas D2 (DRD2, DRD3, DRD4) receptors inhibition [5]. Although both D1 and D2 receptors are found in different brain regions, their effects have been extensively studied in the context of the nucleus accumbens (NAc), where neurons predominantly express the two most common DRD1 and DRD2 types. In this region, dopamine-sensitive neurons contribute to distinct aspects of reward processing and addiction-related behaviors [6, 7]. Moreover, on the population level, they seem to be opposingly modulated by drugs of abuse or sucrose, with DRD1(+) cells activated and DRD2(+) inhibited. However, the modulatory effects of these receptors in other brain regions remain poorly understood.

In this study, we focused on another brain structure regulated by dopamine that plays a crucial role in processing emotional and motivational aspects of experiences – the amygdala. Notably, studies differentiate amygdala nuclei that integrate various sensory and emotion-related information to shape appropriate behavioral responses. For example, neuronal ensembles and synaptic plasticity within the central amygdala (CeA) have been linked to processing and the formation of both aversive and appetitive memories [8–16]. Recent studies have aimed to further disentangle the functional roles of these amygdala subdivisions as well as their specific neuronal subpopulations in various behaviors [8, 14, 17–21].

In this study, we focused on the DRD1(+) and DRD2(+) populations of the CeA, dopamine-sensitive neurons, which remain largely unexplored but are of potential interest given the modulatory effects of the dopaminergic system in processing emotionally relevant stimuli. For example, DRD2(+) cells in the CeA were shown to be a fear-supporting and pain-processing population in mice, making it a specific target in pain-relief-oriented studies [22, 23]. DRD1(+) cells in the medial amygdala, on the other hand, were shown to participate in approach/avoidance behaviors towards predator odors [24]. Specifically, we examined the medial part of the CeA (CeM), which is the primary output nucleus of the amygdala complex, where we found that both DRD1(+) and DRD2(+) cells reside.

The primary aim of this study was to determine whether these two dopamine-sensitive neuronal populations within the CeM are differentially engaged by emotionally salient stimuli of opposing valence: pharmacological reward (cocaine), natural reward (sucrose), and an aversive tastant (quinine). We selected cocaine and sucrose as they represent two distinct types of appetitive rewards, which in NAc engage different neuronal ensembles [25]. They also have different mechanisms of action, as cocaine is a pharmacological reinforcer that strongly activates the dopaminergic system through inhibition of dopamine reuptake, and sucrose is a natural, palatable reward with innate motivational value. This allowed us to compare how dopamine-sensitive neurons in the CeM respond to both drug-induced and naturally driven reward signals. In contrast, quinine (bitter in taste, which is evolutionarily linked to toxin avoidance) was chosen as an aversive tastant to assess how these neuronal populations respond to negatively-valenced stimuli [26, 27]. We hypothesized that DRD1(+) and DRD2(+) neurons would exhibit distinct activation profiles depending on the stimulus type, similar to their roles in other brain regions such as the NAc [28].

Thus, using in vivo two-photon microscopy, we recorded the calcium activity of CeM neurons in mice exposed to these stimuli.

Our results indicate that DRD1(+) and DRD2(+) neurons exhibit differential engagement depending on stimulus valence. Cocaine and sucrose predominantly activated DRD1(+) cells, whereas DRD2(+) cells showed suppressed activity following cocaine and sucrose exposure but were preferentially engaged by the aversive quinine stimulus. These findings suggest that while both dopamine-sensitive populations participate in the processing of motivationally significant stimuli, they do so in distinct and opposing ways. Furthermore, we show that cells responding to different stimuli display a range of activity patterns, with some increasing and others decreasing their activity, highlighting the complexity of the responses of dopamine-sensitive neurons in the CeM.

In addition to performing the analysis of calcium activity of the CeM neurons, we also used an infrared camera to monitor facial expressions triggered by these stimuli. It allowed us to find the behavioral outcomes of restrained animals exposed to cocaine, sucrose, and quinine. Such outcomes are well-characterized in freely moving animals and allow for assessing, for example, the stimulus preference, drug pharmacodynamics, and effects of intervention (e.g., transgenic modifications) that alter responses to stimuli. Here, using advanced machine learning techniques, we tracked faces of head-fixed mice, complementing previously gathered data on quinine and sucrose [26]. We also provide the most detailed facial analysis to date of mice intoxicated with cocaine, revealing patterns surprisingly similar to stimulant effects observed in humans. This analysis offers a novel behavioral readout and could be a new approach for studying cocaine’s effects.

## Materials and methods

### Animals

All experiments were performed on adult DRD1-Cre (B6;129-Tg(Drd1a-cre)120Mxu/Mmjax, RRID: MMRRC_037156-JAX), or DRD2-Cre (B6.FVB(Cg)-Tg(Drd2-cre)ER44Gsat/Mmucd, RRID: MMRRC_032108-UCD) mice of both sexes, 3–5 months old. Mice were housed in individual open-top cages with food and water *ad libitum*. Mice were single-housed after GRIN (Gradient Index) lens implantation to prevent damage to the implant and to ensure proper recovery of animals. The housing room was maintained at 21–23 °C with 40–60% humidity and a 12:12 h light/dark cycle (lights on at 7:00 a.m.), with 15 air changes per hour. Sample sizes were determined using standard power calculations (power = 0.90, α = 0.05), with variance and effect size estimates derived from our previous studies. All studies were performed following the European Council Directive of 22 September 2010 (2010/63/EU), Animal Protection Act of Poland, and approved by the 1st Local Ethics Committee in Warsaw (permission number 1225/2021). All efforts were made to minimize the number of animals and their suffering.

### Viral injections and GRIN lens implantation for calcium imaging

DRD2-Cre or DRD1-Cre mice were anesthetized with isoflurane (induction at 5%, maintenance at 1.1–1.5%) and received a cocktail of painkillers (butorphanol 3 mg/kg, and tolfenamic acid 3 mg/kg). During anesthesia, the skin above the scalp was removed, and the skull was scratched with a scalpel. 200 nl of adeno-associated virus (pGP-AAV-syn-FLEX-jGCaMP8m-WPRE [29] 7 × 10¹² vg/mL, Addgene 162378-AAV1) were stereotactically injected into the right CeM (coordinates from bregma: ML + 2.2, AP -1.3, DV 4.6 mm) at a rate of 75 nl/min. During the same procedure, a GRIN lens (Inscopix GLP-0673; 7.3 mm×0.6 mm) was implanted above the CeA (coordinates from bregma: ML + 2.2, AP -1.3, DV 4.5 mm). A GRIN lens was inserted into the brain using a holder (Mightex HLR-GRIN-050) at a rate of approximately 0.1 mm/min, with special care taken to prevent bleeding. After implantation, the sides of the GRIN lens and the skull were covered with surgical glue (Surgibond). Then, dental cement was used to mount a head plate (Neurotar NTR000484-06) on the animals’ heads. The surface of a GRIN lens was covered with a sealant (World Precision Instruments Kwik-Sil). After surgery, mice received painkillers and antibiotics (butorphanol 3 mg/kg, enrofloxacin 5 mg/kg, and tolfenamic acid 3 mg/kg) subcutaneously for the next 5 days. Before any further habituation, mice were allowed to recover for three weeks.

### Two-photon calcium imaging

Before imaging sessions, mice were habituated to head-fixation procedures and exposure to white noise (60 dB). Before each head-fixation session, mice were lightly anesthetized with isoflurane and head-fixed in a chamber. Habituation was conducted gradually, starting with only 1 min of head-fixation in darkness. After a week of habituation, imaging sessions started. For five consecutive days, mice were head-fixated, and once the focal plane on a GRIN lens was identified, mice were fully awakened. They were injected subcutaneously with cocaine dissolved in saline (90–110 μl; final concentration 20 mg cocaine/kg body weight) or the same volume of saline alone. Imaging was conducted immediately after the injection. During imaging, a mouse’s face was illuminated with an infrared diode and recorded using an infrared camera (Basler acA2000-165umNIR). The locomotor activity of a mouse was tracked using an encoder that measured rotations of the rotating disc via a Teensy microcontroller (Teensy 3.5 ARM Cortex M4). The infrared camera and an encoder were synchronized with the two-photon microscope, where acquired frames served as a master clock for the other devices.

For most mice, an additional day of imaging was conducted, during which they were exposed to a 7.5% (weight/volume) water sucrose solution and 0.1 mM quinine dissolved in water. Both solutions were delivered through a custom-made lickport as 2.5 μl droplets (calculated based on the diameter of the droplet on the movie; 1.8 mm). Liquids were delivered at random intervals, but not shorter than 60 s. Each session began with 4–6 trials of sucrose to encourage initial engagement with the spout. After this, quinine trials were introduced, and the subsequent sucrose and quinine trials were randomly intermixed. Across animals, the number of licking responses per session ranged from 6 to 10 for sucrose and from 2 to 4 for quinine. Liquid delivery pumps (Velleman WPM447) were used (generating 40 dB noise, which did not exceed the 60 dB white noise played in the background). After each solution delivery, the Arduino operating a pump sent a TTL signal about the delivered solution to the data stream. Based on these signals, subsequent licks were classified as corresponding to either a quinine or a sucrose droplet.

### Two-photon imaging data analysis

Videos from an infrared camera were cropped to the size of a mouse head using FFmpeg software. These cropped videos were then analyzed with DeepLabCut software, which enabled tracking of several points on the mouse head [30]. These tracking points, along with SimBA [31] software, allowed for the detection of specific facial movements. SimBA software was also used to compute First-Second Time-Triggered Correlation (FSTTC) between face movements in a 2-second time window. Videos of mice’s faces during sucrose exposure were analyzed with BehaView software (BehaView 0.0.23 by Dr. Pawel Boguszewski, Laboratory of Behavioral Models, Nencki Institute of Experimental Biology PAS, Warsaw), where the onsets of droplet licks were manually determined. Based on the recorded signals from the Arduino indicating which solution was delivered, subsequent licks were automatically classified as corresponding to either quinine or sucrose trial.

To improve the signal-to-noise ratio, images from a two-photon microscope were averaged two times, which gave an effective frame rate of 5 Hz. Motion correction, cell segmentation, and extraction of the fluorescence signal were performed using Suite2P software [32]. Subsequently, a custom script written in Matlab was used for the detection of calcium events for individual cells. Specifically, ΔF/F was computed as the relative change in fluorescence intensity over time, using each cell’s average fluorescence as baseline (F₀). Then, ΔF/F traces were subjected to band-pass filtering to reduce both slow drifts and high-frequency noise (4th-order Butterworth low-pass filter with a cutoff at 0.55 Hz, followed by a high-pass filter at 0.006 Hz). To ensure consistent sensitivity across cells with varying baseline activity or signal-to-noise ratios, transient events were detected using a dynamic threshold approach. For each neuron, the high-pass filtered signal was scanned, and a transient was counted when the signal exceeded 40% of the cell’s own maximum response. To calculate the frequency of calcium transients for each neuron in saline/cocaine sessions, the number of detected events was then divided by the total recording time.

The activity data for each neuron were organized into discrete bins. For the cocaine experiment, data were combined into 420 bins (3 s each), with bin 1 starting just after the subcutaneous drug injection. To compare the activity before and after the onset of drug effects, neuronal activity in bins 1 to 120 was considered as pre-drug activity and from bins 160 to 420 as post-drug activity. These bin ranges were selected based on changes in the locomotor activity of mice, with a noticeable shift in velocity occurring around these time points. For each mouse, frequencies of transients during the cocaine experiment were pooled across neurons and days, shuffled 1000 times, and reassigned to five pseudo-sessions matching original neuron counts (similar to van Zessen et al. 2021 [33]). The difference in average frequency between cocaine (Days 2, 3, 5) and saline (Days 1, 4) was compared between observed and shuffled data. For the sucrose/quinine experiment, the activity of neurons was analyzed in 150 bins (0.2 s each, corresponding to 5 Hz frame rate) with 50 bins before and 100 after the lick. Activity measured from bins -50 to -45 was treated as pre-lick activity, and from 5 to 100 as post-lick activity.

The neuronal activity data were later standardized by calculating the z-score for each neuron using the formula:

$${\rm{Z}}-{\rm{score}}=({\rm{x}}-{\rm{\mu }})/{\rm{\sigma }},$$

where x represents the activity at an individual data point, and ${\rm{\mu }}$ and σ refer to the mean and standard deviation of activity during the pre-stimulus period, respectively. Z-score calculation allowed for the standardization of neuronal responses across all sessions, enabling comparisons of activity between pre- and post-exposure periods. To further assess the significance of neuronal responses to sucrose/quinine, we used a time-bin shuffling approach to create a null distribution of z-scores. For each neuron, the 150 time bins of activity were randomly permuted 1000 times, and z-scores were computed relative to the baseline period [34]. The average z-score in the response period was compared to the null distribution. To identify distinct patterns in neuronal responses, we performed Principal Component Analysis (PCA) on the post-cocaine and post-lick activity for each neuron. Using the elbow method, we determined the optimal number of clusters, and subsequently, we performed k-means clustering on the PCA-reduced data to group neurons based on their activity profiles during the post-cocaine or post-lick periods. This unsupervised approach allowed us to identify populations with distinct response patterns without imposing arbitrary thresholds.

### Statistical analysis

Statistical analysis was conducted using GraphPad Prism 9 software. For each group, normality was assessed using the Shapiro-Wilk test. Depending on the distribution, either parametric tests or non-parametric tests were applied. All statistical tests were two-tailed, and significance was set at p < 0.05. All statistical analyses are presented in Table 1 in the supplementary materials.

Correlations between clustered neuronal activity and behavioral features were computed in MATLAB. For cocaine sessions, analyses were performed at the individual-mouse level using time-aligned behavioral and clustered activity traces (420 bins). For each mouse, the average activity of predefined clusters was correlated with each behavioral parameter using Spearman’s rank correlation. Significance was determined using circular time-shift permutations (2000 iterations), generating null distributions for each cluster-behavior pair. For sucrose and quinine sessions, behavioral signals were linearly interpolated to a temporal resolution matching neuronal activity. Behavioral traces were normalized to a pre-reward baseline, and cross-correlations between the mean activity of each predefined cluster and behaviors were computed. Statistical significance was assessed using permutation testing (2000 circular shifts), and empirical p-values were derived from the resulting null distributions.

## Results

### DRD1 and DRD2 are differentially distributed in sub-nuclei of the CeA

Although the central amygdala is often considered a single entity, it is a heterogeneous structure composed of distinct sub-nuclei: medial, lateral (CeL), and capsular (CeC), with specialized functions and diverse neuronal populations [35–37]. These sub-nuclei differ not only in their inputs but also in their cellular composition, characterized by distinct molecular markers. Notably, the CeM and CeL can be distinguished by their anatomical organization, such as projections from the basolateral amygdala (BLA), which demarcate these nuclei. However, no clear anatomical border exists between the CeL and CeC, except when using specific molecular markers. In this study, we focused on dopamine-sensitive neurons, which have previously been identified in the CeA. Specifically, we examined the two major subpopulations of such neurons: DRD1(+) and DRD2(+) cells.

**Fig. 1:**
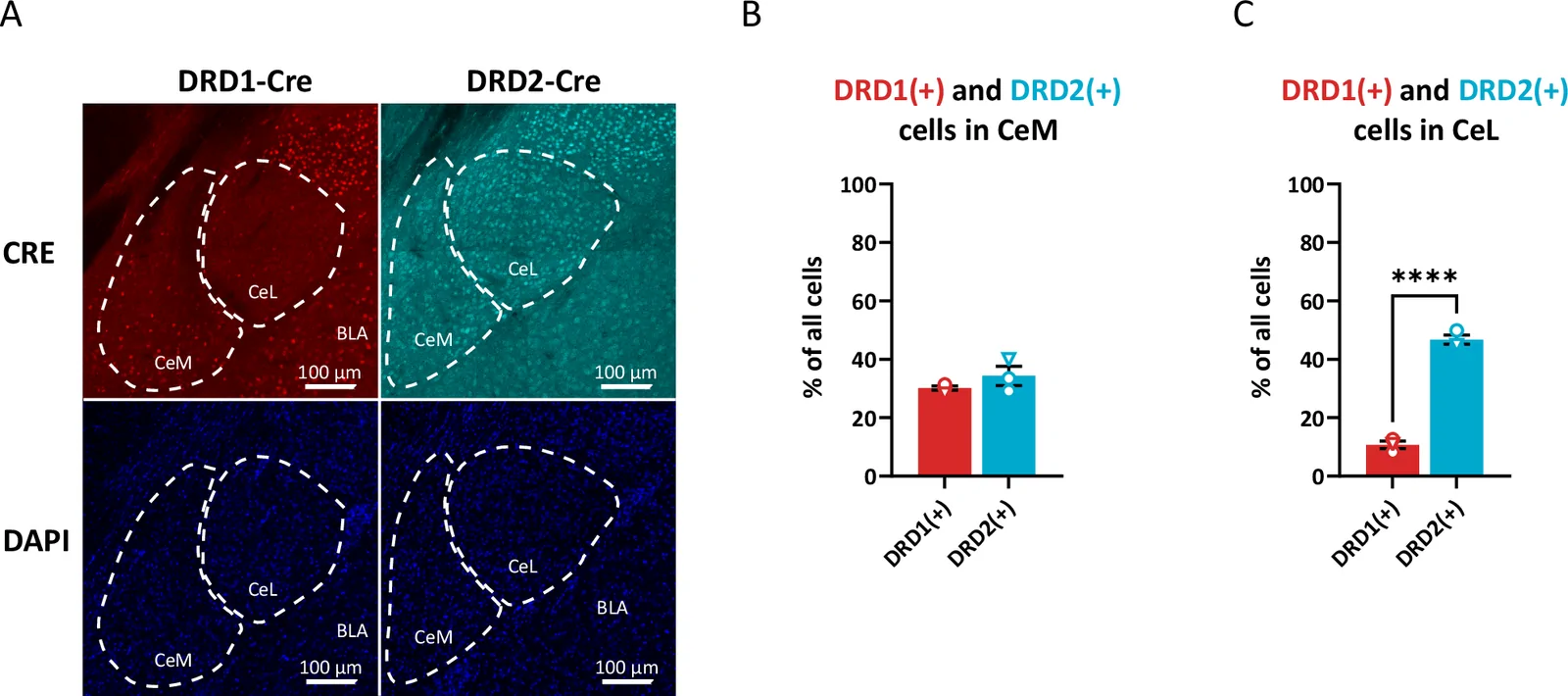
Different patterns of DRD1(+) and DRD2(+) cells in subnuclei of the central amygdala. (**A**) Labelling of Cre protein in brain slices from DRD1-Cre (red) and DRD2-Cre (cyan) mice. Dashed lines represent the borders of CeM and CeL nuclei. (**B, C**) Graphs representing the average number of Cre-positive cells in CeM (**B**) and CeL (**C**) in DRD1- and DRD2-Cre mice. For (**B**) N = 3(14) and for (**C**) N = 3(15), where N = number of animals (number of analyzed images). Statistical differences for (**B**) and (**C**) were measured with paired t-tests, with p-value p < 0.0001 for (**B**) and p = 0.1711 for (**C**).

To determine the distribution of DRD1(+) and DRD2(+) neurons within the CeA, we immunolabeled Cre protein in transgenic DRD1-Cre and DRD2-Cre mice, labeling Cre-expressing neurons (Fig. 1). Our analysis revealed that DRD1(+) and DRD2(+) neurons were not uniformly distributed across the CeA. The CeL (15 brain slices from 3 mice) was predominantly composed of DRD2(+) neurons, whereas the CeM (14 brain slices from 3 mice) contained a more balanced mix of DRD1(+) and DRD2(+) cells (Fig. 1B, C). Notably, the distribution of DRD1(+) and DRD2(+) cells did not allow for a clear distinction between the CeL and CeC.

Dopaminergic signaling may also influence the CeA indirectly through dopamine-sensitive neurons projecting into this region. To investigate this, we performed retrograde tracing by injecting Cre-dependent retrograde viral tracers (pAAV-hSyn-DIO-EGFP or hSyn-DIO-mCherry) into the CeA of DRD1-Cre and DRD2-Cre mice. We then analyzed the distribution of DRD1(+) and DRD2(+) neurons projecting to the CeA using semi-automated structural annotation software [38, 39] (Fig. Supp. 1). Our results showed that the majority of dopamine-sensitive inputs to the CeA originated from DRD1(+) neurons, while DRD2(+) projections were largely restricted to the caudoputamen (CP) and CeA itself. Specifically, these projections originated from the region where the CeC is usually found. In contrast, DRD1(+) neurons projecting to the CeA were identified in multiple brain regions, including layer V of the cortex (somatomotor, primary somatosensory, supplemental somatosensory, prelimbic, infralimbic, retrosplenial, agranular insular, gustatory, and visceral areas), as well as in the CP and zona incerta (ZI).

### Mouse behavior and facial expressions triggered by cocaine, sucrose, and quinine exposure during in vivo two-photon imaging

In this study, we focused on the central medial amygdala, the primary output nucleus of the CeA. The CeM was of particular interest because it contains both DRD1(+) and DRD2(+) neurons, whereas the CeL is predominantly composed of DRD2(+) cells. This distribution made the CeM an ideal structure for investigating how these two neuronal populations respond to rewarding and aversive stimuli.

**Fig. 2:**
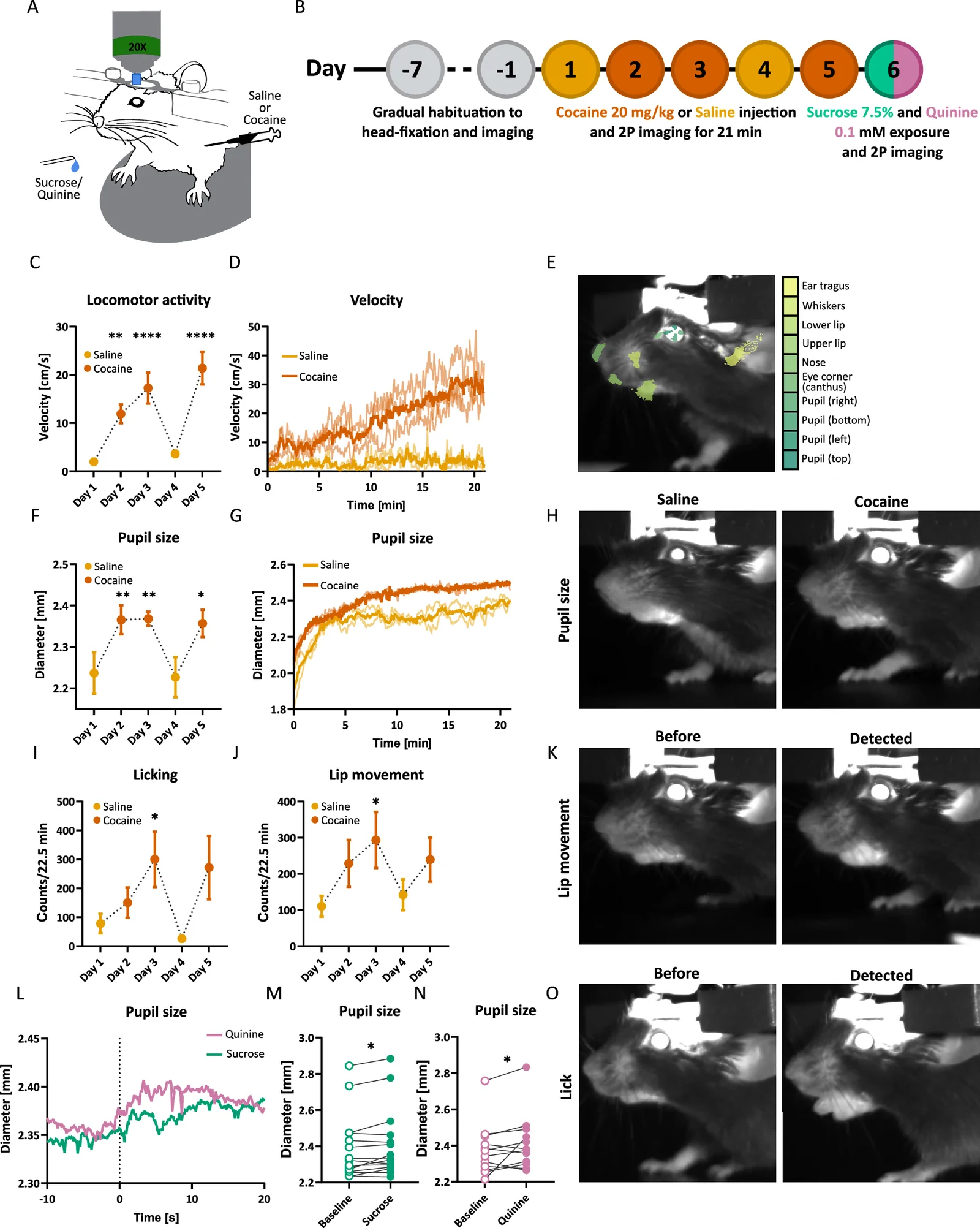
Facial expressions and locomotor activity of head-fixed mice exposed to cocaine, sucrose, and quinine. (**A**) Schematic representation of the mouse under the two-photon microscope. Head-fixed mouse stands on a rotating disc. (**B**) Experiment design. After the habituation period, mice were imaged under a two-photon microscope. Once per day, mice received subcutaneous injections of saline or 20 mg/kg body weight cocaine solution. On the last day of the experiment, mice received droplets of 7.5% sucrose solution and 0.1 mM quinine solution as the last droplet. (**C**) Graph representing the locomotor activity of a mouse on a running disc during cocaine and saline sessions. Data is presented as averages for all mice ± SEM. (**D**) Averaged velocity of mice running on a rotating disc during all sessions: saline (yellow) and cocaine (orange). Lighter lines represent the averaged velocity across individual days. (**E**) Facial features tracked with DeepLabCut software. Colored areas represent the positions of points detected by the software. (**F**) Graph representing pupil diameter during cocaine and saline sessions. The graph represents averages for all mice ± SEM. (**G**) Averaged pupil diameter during saline (yellow) and cocaine (orange) sessions. Lighter lines represent the averaged diameter across individual days. (**H**) Exemplary images of a mouse exposed to saline (left) and cocaine (right) showing differences in pupil dilation (**I, J**). Graphs representing averaged numbers of lip movements (**J**) and licks (**I**) during cocaine and saline sessions. Graphs represent averages for all mice ± SEM. (**K, O**) Exemplary images before and during lip movement (**K**) and licking behavior (**O**). (**L**) Graph representing the averaged pupil size 10 s before and 20 s after a lick of sucrose (green) and quinine (pink). The dotted line represents the onset of a lick. (**M, N**) Graphs showing average pupil size across all trials with sucrose (**M**) and quinine (**N**) for individual mice (triangles for males and circles for females). N numbers in groups: for (**C)** and (**D**): N = 22 (11♂, 11♀) and for (**F, G, I, J)**: N = 17 (8♂, 9♀), where N = number of animals. Number of groups for **L-N**: N sucrose = 18 (10♂, 8♀) and N quinine = 13 (6♂, 7♀), where N = number of animals. Statistical differences for (**C, I, J**) were measured with repeated-measures lognormal one-way ANOVA with Holm-Šídák’s multiple comparisons test, with p values for (**C**) day 1 vs. day 2 p < 0.0001, day 1 vs. day 3 p < 0.0001, and day 1 vs. day 5 p < 0.0001, and p values for (**I**) day 1 vs. day 2 p = 0.00389, and p values for (**J**) day 1 vs. day 2 p = 0.0389. Statistical differences for (**F**) were measured with one-way ANOVA with repeated measures with Holm-Šídák’s multiple comparisons test, with p values for day 1 vs. day 2 p = 0.0326, day 1 vs. day 3 p = 0.0326, and day 1 vs. day 5 p = 0.0326. Statistical differences for (**M**) were measured with the Wilcoxon test, with p values p = 0.0385. Statistical differences for (**N**) were measured with paired t-test, with p value p = 0.0879. Statistical differences are represented by stars, where *, **** correspond respectively to p < 0.05 and p < 0.0001.

To assess CeM neuronal activity in vivo, we developed an imaging protocol during rewarding/aversive stimuli exposure, preceded by a gradual habituation of mice to head fixation (Fig. 2A, B). After the habituation, we first examined the effects of cocaine administration. Mice received subcutaneous injections of either saline or cocaine (20 mg/kg body weight) before each imaging session. We specifically chose the subcutaneous injection, as such was shown to delay the onset of pharmacological effects in the rodent brain [40]. The protocol consisted of one saline session on the first day, followed by two days of cocaine exposure, another saline session, and a final cocaine session. On the last imaging day, we presented mice with liquid rewards via a lickport, delivering a 7.5% sucrose solution followed by an aversive 0.1 mM quinine solution (Fig. 2A, B). During imaging, head-fixed mice stood on a freely rotating disk, allowing us to measure their locomotor activity (Fig. 2C, D). Simultaneously, an infrared camera recorded their facial expressions. Using DeepLabCut, we tracked facial landmarks on mice heads (Fig. 2E), and with SimBA, a supervised machine-learning software [31], we quantified distinct facial movements, including jaw, nose, and ear movements, as well as licking behavior.

In this controlled setting, we identified several behavioral and facial features associated with cocaine intoxication. Mice exhibited significantly greater locomotor activity during cocaine sessions compared to saline sessions, peaking approximately 20 min after injection (Fig. 2C, D). This progressive increase in movement over repeated exposures is characteristic of behavioral sensitization, a hallmark effect of psychostimulants [41]. The most prominent physiological indicator of cocaine’s effect was pupil dilation (Fig. 2F–H).

Facial expression analysis revealed that cocaine exposure induced distinct orofacial behaviors. Mice displayed increased jaw movement and licking behavior (Fig. 2I–K). Further geometric analysis of facial landmarks showed that cocaine altered few facial metrics, such as widening the angles between the mouth, eye, and lip while shortening the distances between the nose, eye, and whiskers (Fig. Supp. 2E–J). Although blinking and whisker movements were also observed, they were less consistent indicators of cocaine intoxication. However, nose movements were significantly more frequent during cocaine sessions (Fig. Supp. 2A–D).

We next examined facial expressions during sucrose and quinine consumption. Using BehaView software, we manually identified onsets of licking events and analyzed facial movements from 10 s before to 20 s after the onset of each lick. Interestingly, both sucrose and quinine intake led to pupil dilation, but the effect lasted longer after sucrose consumption (Fig. 2L–O). Additionally, the distance between the eye and whiskers decreased immediately after licking, with this contraction persisting longer for sucrose than for quinine (Fig. Supp. 2K, L, O, P). Averaged analysis of facial features, including the distance between the eye and whiskers, the angles between the eye and the mouth/lip, and perturbations in the average trace of the eye–nose distance (Fig. Supp. 2L, N, R), also allowed for the estimation of the length of the licking behavior, which typically lasted approximately 7.7 s.

To assess how cocaine affects the sequencing of facial behaviors, we used First-Second Time-Triggered Correlation (FSTTC) to compute pairwise temporal associations between all detected behaviors in a 2 s window (Fig. Supp. 3). The analysis revealed a consistent pattern of behavioral transitions across both the saline and cocaine exposures, particularly involving the lick, lip movement, and whiskers movement, which showed moderate positive correlations in both conditions. Mild suppressions of certain transitions, such as those involving blinking and nose movement, were present in both groups, though more pronounced in the cocaine group. Compared to saline exposure, mice under cocaine showed altered behavioral coupling, particularly stronger temporal linkage between nose and whisker movements, and reduced suppression of blink transitions.

### Opposing activation of DRD1(+) and DRD2(+) in the CeM cells following cocaine exposure

**Fig. 3:**
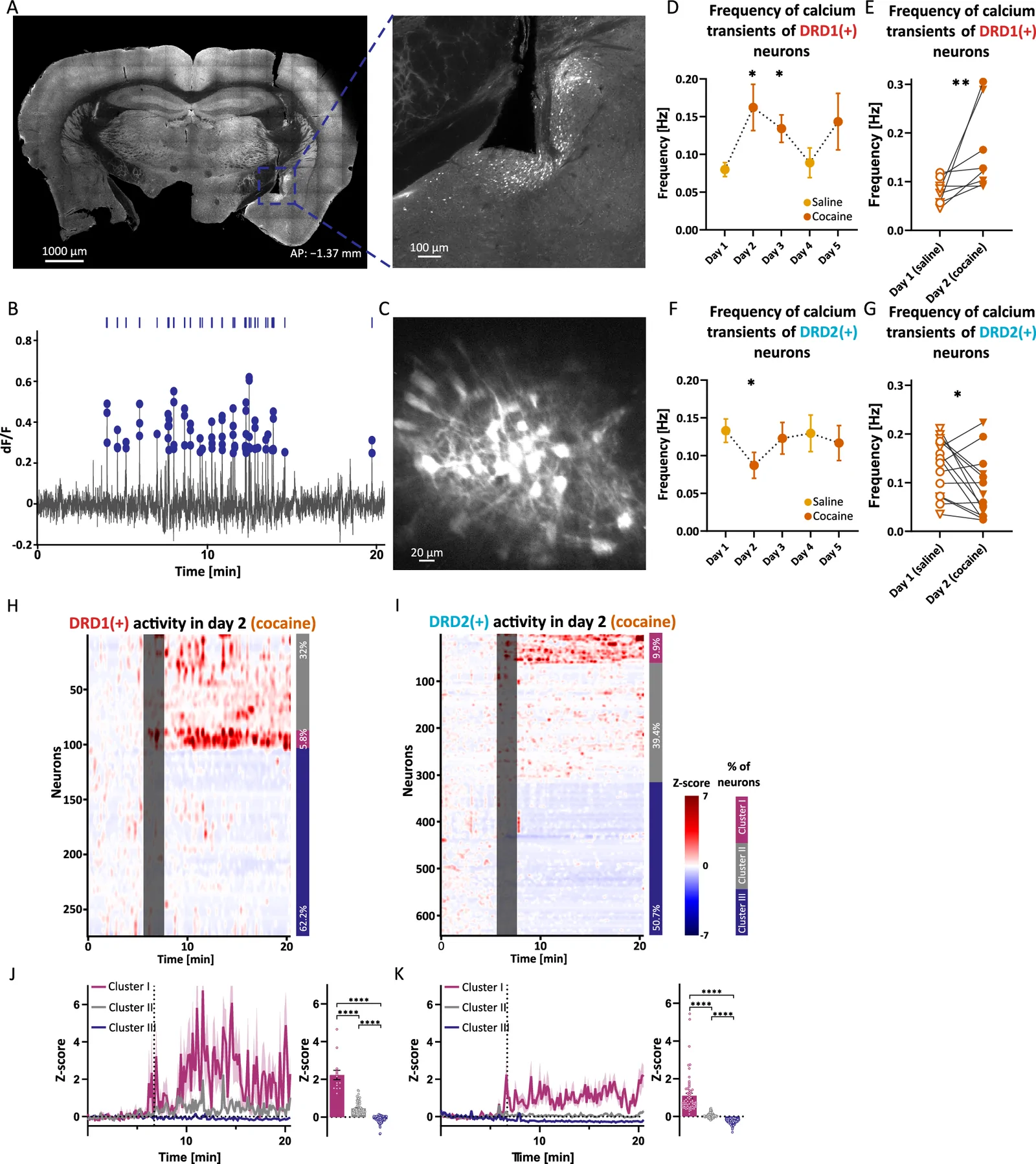
Calcium activity of DRD1(+) and DRD2(+) neurons in the medial part of the central amygdala during cocaine exposure. (**A**) Example image of the scar in the brain after GRIN lens implantation. Cells expressing GCaMP8m are shown in white. AP in the lower-right corner indicates the anterior-posterior position (in mm) relative to bregma. (**B**) Representative calcium trace with detected events. (**C**) Example image from a two-photon microscope of GCaMP8m-expressing neurons in the CeM, visible through a GRIN lens. (**D, F**) Graphs showing the frequency of calcium events detected in DRD1(+) (**D**) and DRD2(+) (F) neurons across all sessions. Graphs represent averages of events for all mice ± SEM. **(E, G**) Graphs showing the averaged frequency of calcium events in DRD1(+) (**E**) and DRD2(+) (**G**) neurons during the first saline (day 1) and first cocaine (day 2) injections. Graphs represent individual averages of events for each mouse (triangles for males and circles for females). (**H, I**). Heatmaps showing calcium activity of all recorded neurons during the first cocaine injection (day 2) for DRD1(+) (**H**) and DRD2(+) (**I**) neurons. For each neuron, the activity was binned into 3-second intervals, and a z-score was calculated based on their activity before and after 7 min of recording (time indicated by dotted line). Cells were clustered with PCA on binned neuronal activity, followed by k-means clustering on the PCA-reduced data to group neurons based on their activity to form three clusters. (**J, K**) Graphs representing the mean activity of clusters of DRD1(+) **(J**) and DRD2(+) (**K**) cells. On graphs, dotted lines represent the 7-minute time used to differentiate baseline and response activity. On the right of the graph, the mean responses of clusters to cocaine are shown. For DRD1(+) N = 8 (5♂, 3♀) and for DRD2(+) N = 14 (6♂, 8♀), where N = number of animals. Statistical differences for (D) and (F) were measured with repeated-measures lognormal one-way ANOVA with Holm-Šídák’s multiple comparisons test, with p values for (D) day 1 vs. day 2 p = 0.0485, day 1 vs. day 3 p = 0.0485, and p value for (F) day 1 vs. day 2 p = 0.0251. Statistical difference for (E) was measured with the Wilcoxon test, with p-value p = 0.0078. Statistical difference for (G) was measured with paired t-tests, with p-value p = 0.0270. Statistical differences for (J) and (K) were measured with two-way ANOVA with Holm-Šídák’s multiple comparisons test, with p values for (J) Baseline vs. Response for Cluster I: p < 0.0001; for Cluster II p < 0.0001; for Cluster III p = 0.0002, and for (K) Baseline vs. Response for Cluster I: p < 0.0001; for Cluster II p = 0.0043; for Cluster III p < 0.0001. Statistical differences are represented by stars, where *, **, ***, **** correspond respectively to p < 0.05, p < 0.01, p < 0.001 and p < 0.0001.

We imaged the calcium activity of dopamine-sensitive neurons in the CeM of head-fixed DRD1-Cre and DRD2-Cre mice through a GRIN lens. In a cre-dependent manner, we expressed a fluorescently labeled calcium indicator (GCaMP8m) in the CeM. We implanted a GRIN lens above the CeM, serving as an optical relay lens, allowing imaging neurons located deep inside the brain. (Fig. 2A). Mice were subcutaneously injected with either saline or cocaine, and the calcium activity was imaged for 21 min at 30 Hz, averaged to 10 Hz. In mice with post-mortem validated expression of cre-dependent GCaMP8m and accurate GRIN lens localization, we performed further analysis of calcium events (Fig. 3A–C, Fig. Supp. 4).

Our results revealed opposing cocaine-induced activity changes in DRD1(+) and DRD2(+) neurons (Fig. 3D–G). The strongest change was observed during the first cocaine exposure, DRD1(+) neurons exhibited a marked increase in calcium transient frequency, whereas DRD2(+) neurons showed a significant decrease (Fig. 3E, G). While DRD1(+) activity remained elevated across subsequent cocaine sessions, DRD2(+) activity was only affected during the first exposure, suggesting a desensitization effect in this population (Fig. 3D, F; Fig. Supp. 5A, B). Overall, the increase of calcium transients frequency of DRD1(+) cells was consistent across sessions, leading to a statistically significant difference between the average activity during all saline and all cocaine sessions, as well in comparison to shuffled data. In contrast, DRD2(+) neurons did not exhibit a sustained change in activity across the remaining sessions, resulting in no statistically significant differences when comparing saline to cocaine sessions or to shuffled controls (Fig. Supp. 5E, F).

Cocaine’s pharmacological effects following subcutaneous injection emerged progressively, with an inflection point around 7 min post-injection, as determined by a consistent increase in locomotor activity and pupil dilation across animals (Fig. 2D, G). This behavioral shift served as a reference point to align calcium imaging data. We sorted all recorded neurons based on their activity profiles before and after this 7-minute mark (Fig. Supp. 5A, B). Cells exhibited heterogeneous response patterns during cocaine intoxication, with some increasing their activity, others decreasing it, and some showing no change in activity. To quantify these dynamics, we calculated the z-score of each neuron’s activity relative to a baseline period before the 7-minute time point (Fig. Supp. 5, D). At the population level, these opposing responses largely cancelled each other out, resulting in a minimal net change in average activity (Fig. Supp. 5G, H). Thus, to differentiate functionally distinct neuronal populations responding to cocaine, we used principal component analysis (PCA) and k-means clustering to identify three distinct response categories within each neuronal population for the first response to the drug (Fig. 3H–K). Among DRD1(+) neurons, the largest fraction (62%) was a mix of low and non-responsive, while the remaining cells exhibited varying degrees of increased activity. In contrast, the majority of DRD2(+) neurons (50.7%) significantly decreased their activity following cocaine exposure, while a smaller subset (~11.7%) exhibited moderate or low (39.4%) activation.

These findings suggest that DRD1(+) and DRD2(+) neurons in the CeM respond to cocaine in a highly divergent manner, with DRD1(+) cells predominantly activated and DRD2(+) cells largely suppressed. Moreover, the transient nature of DRD2(+) inhibition suggests that this population may play a key role in early-stage cocaine processing, while DRD1(+) neurons remain persistently engaged across repeated exposures.

To measure associations between cluster-specific neuronal dynamics and facial and locomotor behaviors during cocaine exposure, we computed Spearman correlations between binned cluster activity and binned occurrence of previously quantified behavioral parameters (Fig Supp. 6). Overall, correlation strengths were modest, with mean coefficients not exceeding −0.25 or 0.25 across conditions. In DRD1(+) mice, cluster II, characterized by moderate activation following cocaine administration, showed the strongest associations with behavioral measures. The most prominent correlation was observed with pupil size (ρ = 0.202, p < 0.001), although the magnitude of this relationship remained moderate (Fig. Supp. 6A).

In DRD2(+) mice, clusters I (cocaine-activated cells) and III (cocaine-inhibited cells) exhibited the strongest behavioral associations (Fig. Supp. 6A). Notably, these two clusters were correlated with largely overlapping behavioral parameters but in opposite directions, reflecting their differential response profiles. Correlations were observed for locomotor activity, pupil size, whisker movements, distance between whiskers and nose, and the angle between the eye–mouth and eye–lip. However, as in DRD1(+) mice, the average correlation coefficients did not exceed -0.25 or 0.25, indicating that the relationships, while statistically significant, were relatively weak in magnitude.

### Opposite regulation of DRD1(+) and DRD2(+) neurons in the CeM upon sucrose and quinine exposure

**Fig. 4:**
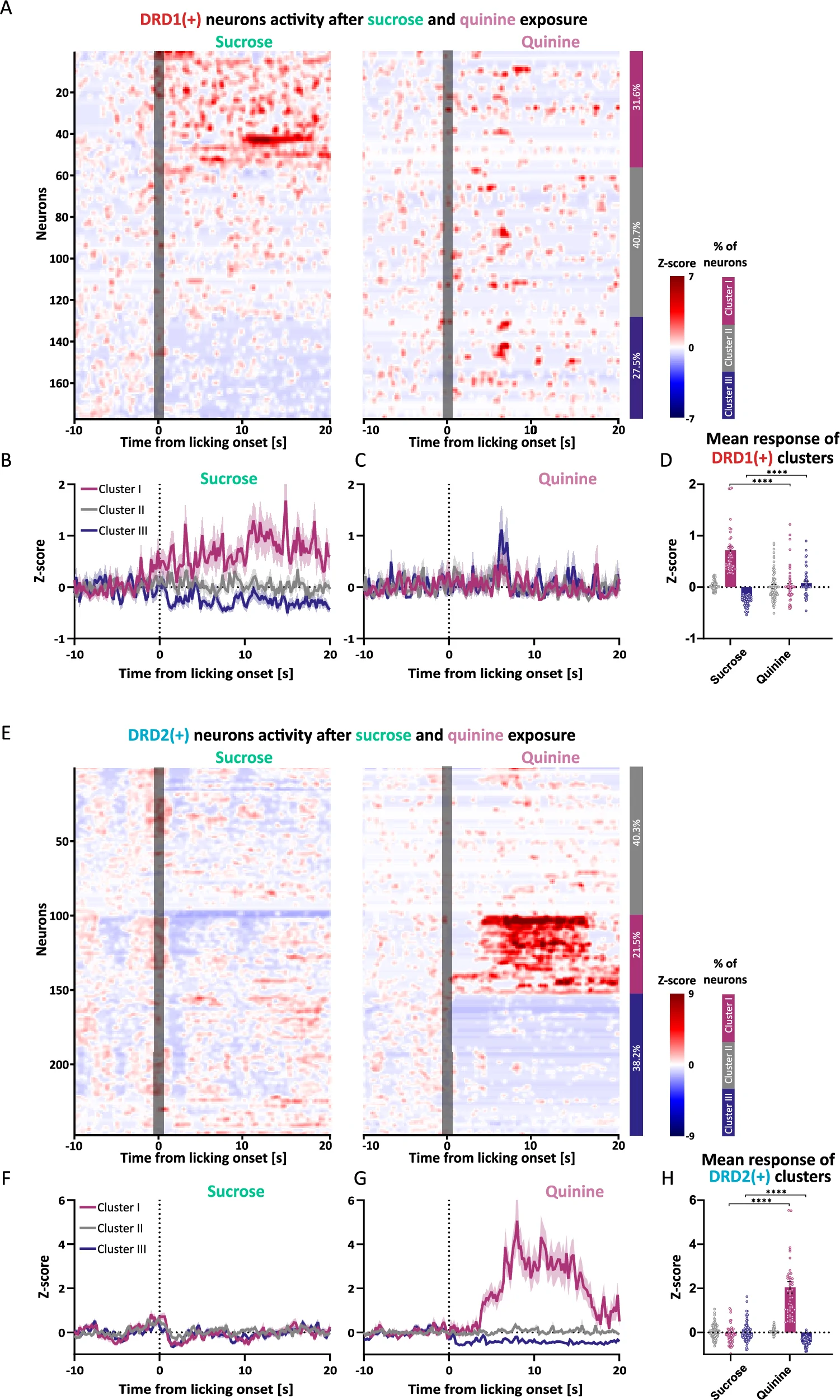
Different populations of DRD1(+) and DRD2(+) neurons in the central medial amygdala are activated after sucrose and quinine exposure. (**A, E**) Heatmaps showing calcium activity of all recorded DRD1(+) (**A**) and DRD2(+) (**E**) neurons. Averaged activity of neurons across all trials for 7.5% sucrose solution and 0.1 mM quinine exposure. Z-scores for responses to sucrose and quinine were calculated for each neuron. Cells were clustered using PCA on the neuronal responses to sucrose and quinine, followed by k-means clustering on the PCA-reduced data to group neurons based on their activity into three clusters. The onset of licks is represented as a dotted line. (**B, C, F, G**) Plots representing the averaged responses of clustered neurons. The dotted line represents the onset of a lick. (**D, H**) Mean responses of DRD1(+) (**D**) and DRD2(+) (**H**) clusters to sucrose and quinine. Numbers of animals in groups for DRD1(+) N = 6 (3♂, 3♀), and for DRD2(+) N = 8 (4♂, 4♀). Statistical differences for (**D**) and (**H**) were measured with two-way ANOVA with Holm-Šídák’s multiple comparisons test, with p values for (**D**) for sucrose Baseline Cluster I vs. Response Cluster I: p < 0.0001; Baseline Cluster II vs. Response Cluster II: p = 0.0706; Baseline Cluster III vs. Response Cluster III: p < 0.0001, and for (**H**) for Quinine Baseline Cluster I vs. Response Cluster I: p = 0.0140; Baseline Cluster II vs. Response Cluster II: p < 0.0001; Baseline Cluster III vs. Response Cluster III: p < 0.0001. Statistical differences are represented by stars, where **** correspond respectively to p < 0.0001.

Even more robust effects were observed when we evaluated the activity of DRD1(+) and DRD2(+) cells responding to natural appetitive and aversive stimuli – sucrose and quinine in animals naive to these tastants. We measured DRD1(+) and DRD2(+) cell activity 10 s before and 20 s after the onset of lick and calculated the z-score of each cell based on their activity before the lick (Fig. 4; Fig. Supp. 7).

Similar to cocaine responses, DRD1(+) and DRD2(+) neurons displayed diverse activity patterns—some cells increased their activity, some decreased, and others remained unchanged. This heterogeneity masked the overall mean effect; however, on average, DRD1(+) neurons exhibited increased activity following sucrose consumption, while DRD2(+) neurons showed a decrease (Fig. Supp. 7; Fig. Supp. 8A–D). Moreover, DRD2(+) showed a pattern with a peak just before the lick and a dip immediately after. During quinine trials, we observed a strikingly different pattern: only DRD2(+) neurons increased their activity, whereas DRD1(+) neurons showed no significant change (Fig. Supp. 8E–H).

Since sucrose and quinine were presented within the same imaging session, we could directly compare the responses of individual neurons to both stimuli. Using principal component analysis (PCA) and k-means clustering, we categorized cells into functional subgroups based on their activity patterns (Fig. 4). DRD1(+) neurons predominantly responded to sucrose, forming clusters that either increased or decreased their activity upon exposure (Fig. 4A–D). Interestingly, in all clusters of DRD1(+) neurons during quinine exposure, we observed that the peak of activity was around 8 s after the onset of licking, which corresponds to the moment shortly after the mouse terminates its licking behavior, which, based on our behavioral analysis, typically lasted approximately 7.7 s. This post-licking activity may reflect a neural response associated with the aversive evaluation of the stimulus or the behavioral transition following stimulus consumption. In contrast, DRD2(+) neurons primarily responded to quinine, with distinct clusters showing either activation or suppression (Fig. 4E–H). Notably, a substantial fraction of neurons, 40.7% of DRD1(+) and 40.3% of DRD2(+), fell into clusters that did not respond significantly to either stimulus (Fig. 4).

To quantify both the strength and temporal structure of associations between cluster-level neuronal dynamics and facial behavior during sucrose and quinine exposure, we performed cross-correlation analyses between cluster activity and behavioral parameters (Fig. Supp. 9). This approach allowed us to assess not only the strength of the relationship but also its temporal structure, distinguishing whether behavioral changes preceded cluster activity (maximum correlation at lag < 0) or followed it (when maximum correlation at lag > 0).

In DRD1(+) mice during quinine exposure, all clusters exhibited relatively weak correlations with facial parameters (correlation coefficients < 0.5; Fig. Supp. 9A). In contrast, during sucrose exposure, several facial features showed strong associations with specific clusters. In particular, pupil size, the distance between the eye and whiskers, and the the eye-mouth/eye-lip angle displayed pronounced positive or negative correlations (coefficients > 0.5 or < -0.5) with clusters I and III. The strongest correlations occurred within a temporal window ranging from -1.4 to 1.4 s, depending on the specific cluster-behavior pair, indicating tight temporal coupling.

For DRD2(+) mice, the pattern differed markedly. Here, correlations were generally stronger during quinine exposure than during sucrose presentation (Fig. Supp. 9B). In addition to pupil size and eye-mouth/eye-lip angle, the distance between the eye and nose emerged as a highly correlated parameter. Clusters I and II showed the most consistent associations. Pupil size exhibited the strongest correlations with these clusters, occurring with little lag (0 s for cluster III and 1.4 s for cluster I). Notably, for DRD2(+) cells, behavioral changes more often followed cluster activity, with long positive lags ranging from 5.4 to 12 s. An exception was observed for the relationship between cluster I activity and the eye-mouth/eye-lip angle, where behavioral changes preceded neuronal activity (lag = -3.4 s).

These findings suggest that DRD1(+) neurons in the CeM are primarily engaged in processing appetitive stimuli, whereas DRD2(+) neurons predominantly respond to aversive stimuli. The distinct temporal dynamics of DRD2(+) neurons, particularly their pre-lick peak during sucrose trials and post-lick activation during quinine exposure, highlight their potential role in encoding stimulus valence and guiding behavioral responses. It also indicates differences in the temporal coordination between neuronal cluster dynamics and fine-scale facial features, suggesting distinct patterns of sensorimotor integration in DRD1(+) and DRD2(+) populations.

## Discussion

In this study, we primarily found that: (I) DRD1(+) and DRD2(+) neurons in the CeM respond differently to both rewarding and aversive stimuli. (II) Dopamine-sensitive cells in the CeM exhibit diverse response profiles, with some subpopulations activated and others inhibited by the same stimuli. (III) Mouse facial expressions reliably reflect cocaine intoxication, and these changes resemble facial patterns previously observed in humans.

### CeM DRD1(+) and DRD2(+) cells function in appetitive and aversive stimuli processing

CeA subpopulations were studied for different behaviors, including foraging. For example, Somatostatin (SST)- and protein kinase C-delta (PKC-δ)-expressing neurons in the CeA, were shown to promote food consumption via a positive-valence mechanism and encoding environmental cues during foraging. Specifically, both SST(+) and PKC-δ(+) neurons were responsive during eating, but PKC-δ(+) neurons were essential for learning [18]. Furthermore, serotonergic receptor 2A (Htr2A)-expressing neurons promote feeding and are activated by eating behavior [17, 21].

The role of D1- and D2-expressing neurons in the amygdala has been studied predominantly using dopamine receptor agonists and antagonists delivered through implanted cannulas. However, these techniques impact the entire amygdala complex rather than isolating its individual subdivisions [42, 43]. In the context of feeding behavior, research has shown that activation of D2-expressing cells in the amygdala reduces food intake and sucrose operant conditioning in rats, while the activation or inhibition of D1-expressing cells does not appear to significantly affect these behaviors [44]. On the other hand, blocking D1 receptors in the CeA has been shown to reduce orexin-enhanced saccharin preference, a behavior associated with reward processing [45]. In contrast, the activation of D2-expressing cells in the CeA has been linked to the reward-enhancing properties of pain relief. As the levels of D2 receptors decrease, activating these cells with an agonist can reverse this pain-relieving effect [22]. Additionally, dopamine-sensitive cells in the CeA have been shown to play a critical role in cocaine withdrawal, where a prolonged abstinence period (2 weeks) increases D1 receptor levels in the central lateral subdivision of the amygdala [46]. Importantly, activation of D2 receptors (but not D1) has been shown to reduce cocaine self-administration and the reinstatement of seeking behavior [42].

Our study demonstrated that DRD1(+) and DRD2(+) neurons in the CeM exhibit distinct and often opposing responses to rewarding and aversive stimuli. Cocaine and sucrose exposure predominantly activated DRD1(+) neurons while suppressing DRD2(+) neurons, whereas quinine activated DRD2(+) neurons. During cocaine exposure, the increase in DRD1(+) neuron activity persisted across repeated sessions, while DRD2(+) neuron suppression was observed only during the initial exposure. This suggests that DRD2(+) neurons may be particularly sensitive to novelty or unexpected aversive aspects of the first cocaine intoxication. The absence of this response in later sessions could, however, also reflect circuit-level adaptation, habituation, or compensatory changes in inhibitory networks following repeated drug exposure. In contrast, DRD1(+) neurons more consistently showed a stable increase in activity, possibly by being associated with the reinforcing effects of the drug. Similarly, sucrose exposure activated DRD1(+) neurons, while DRD2(+) neurons showed a transient decrease in activity, particularly just after licking. In contrast, quinine exposure selectively activated DRD2(+) neurons, with no significant involvement of DRD1(+) neurons, suggesting that DRD2(+) cells are more engaged in processing aversive stimuli.

Dopamine signaling plays a key role in highlighting the behavioral relevance of novel stimuli. Activity of dopamine neurons in both the ventral tegmental area (VTA) and the substantia nigra pars compacta (SNc) has been linked to stimulus novelty and shown to diminish as learning progresses [47, 48]. Such learning-dependent adaptations can shape downstream circuit activity, and in the NAc, this effect appears to be receptor-specific: DRD2(+) neurons, but not DRD1(+) neurons, adjust their responses during learning [49]. Consistent with this framework, in the CeM, we observed changes in DRD2(+) neuron activity only during the first cocaine exposure and following quinine exposure during the licking session, which could suggest an enhanced sensitivity of these neurons to novel or salient stimuli. However, sucrose exposure, despite being novel to the animals, elicited only a mild DRD2(+) neurons response, indicating that novelty alone is insufficient to drive robust activation of those cells. This suggests that DRD2(+) neurons’ activity in the CeM is not invariably linked to novelty and may instead be stimulus-specific. Whether and how these neurons adapt as learning progresses remains to be determined using extended behavioral paradigms, such as cue-based tasks.

Our study emphasizes the role of DRD1(+) and DRD2(+) neurons in processing distinct aspects of reward and aversion and aligns with research in other brain regions, such as the NAc, where DRD1(+) neurons increase their activity in response to rewarding stimuli, while DRD2(+) neurons are typically suppressed [28]. In the CeM, we observed similar patterns of activity changes in response to cocaine exposure, reinforcing the idea that these two dopamine-sensitive populations have opposing roles in reward and aversion processing across multiple brain regions.

Interestingly, within both DRD1(+) and DRD2(+) populations, we observed variability in cellular responses to stimuli. Some cells responded with increased activity, while others exhibited decreased activity, suggesting that there may be additional subpopulations within these groups. This variability calls for further investigation into the potential existence of more refined subtypes of DRD1(+) and DRD2(+) neurons, similar to findings in the CeA SST(+) cells and DRD1(+) cells in the NAc, where distinct subpopulations of neurons were found to exhibit different response patterns to cocaine exposure or stimuli of different valence [20, 33, 50]. These subpopulations could provide more nuanced insights into the complex roles of dopamine receptor-expressing neurons in the processing of reward, addiction, and aversion. Specifically, DRD2(+) were shown to co-express or lack proenkephalin or SST(+) and SST(-) among DRD1(+) [14, 37]. Future studies investigating these subtypes may reveal critical information regarding the neural circuits involved in these behaviors, providing valuable targets for therapeutic interventions in addiction and other reward-related disorders.

To gain a more comprehensive understanding of amygdala function, future studies should also explore its other subnuclei, where dopamine-sensitive neurons are present. In our analysis, we also identified such neurons in the CeL, which is predominantly composed of DRD2(+) cells. This contrasts with the CeM, where both DRD1(+) and DRD2(+) neurons are found. Previous studies examining DRD1 and DRD2 expression have reported similar differences between these nuclei [23, 37, 51–54]. However, some studies have shown conflicting patterns, which may be explained by differences in the methods used for identifying receptor expression (transgenic animals versus gene expression) and classifying amygdala subregions, particularly the anatomical division based on BLA projections into the CeA versus classification relying solely on brain atlas coordinates. Based on studies utilizing different transgenic reporter mouse lines and in situ hybridization, overall, in the CeM, DRD1(+) and DRD2(+) cells form non-overlapping neuronal populations, with only a minor fraction (less than 4%) of DRD1(+)/DRD2(+) double positive cells [23, 54]. In other amygdalar nuclei, such as CeL, only 1.8% of cells overlap [54], while in the BLA, 8.95% of DRD1(+) cells are DRD2(+), and 16.12% of DRD2(+) cells overlap with DRD1(+) [55]. Whether these overlapping populations have a specific role in motivated behaviors remains to be elucidated.

Moreover, to fully understand the influence of the dopaminergic system on the amygdala, it is important to investigate the projections of dopamine-sensitive neurons into this region. This is particularly relevant because drugs that modulate dopamine levels may not affect the amygdala directly, but rather through such projections. In our study, we used retrograde tracing to map the origins of dopamine-sensitive inputs to the CeA. We found that DRD1(+) projections originated predominantly from distant areas, including the CP, ZI, somatomotor and somatosensory cortices, agranular insular and visceral areas, prelimbic and infralimbic cortices, retrosplenial area, and gustatory cortex. In contrast, DRD2(+) projections were largely local, arising mainly from the CP and the CeA itself. These findings suggest that DRD1(+) cells in the CeM may integrate broader cortical and subcortical signals, possibly reflecting the motivational and sensory context of stimuli, whereas DRD2(+) neurons might be more locally modulated, perhaps supporting feedback or intra-amygdala processing. Notably, local DRD2(+) projections within the CeA also revealed the presence of the CeC, a subregion that can be easily overlooked due to the lack of clear anatomical separation from the CeL. Further studies are needed to delineate the functional roles of these circuits and their synaptic connections within the CeM.

### Facial expressions and behavior analysis

We performed a comprehensive analysis of mice’s facial expressions in response to sweet and bitter water or cocaine exposure. Similar analyses have been previously done for bitter and sweet water exposure and other stimuli, including painful electric shocks, or unexpected sounds, and were successfully used as a readout of the internal states of animals [26, 56, 57]. For instance, exposure to sucrose and quinine elicited similar facial expressions in mice, primarily reflected in ear positioning and to a lesser extent as a grimace in the lower frontal part of the head [26]. Our study revealed that both sucrose and quinine induced pupil dilation, which is a response commonly recognized as a metric of animal arousal and uncertainty [56, 58]. In our research, pupil dilation was also the most pronounced indicator of cocaine intoxication, an effect known to be triggered by stimulants in humans and used as a rapid screening tool for cocaine use [59]. Pupil dilation was also observed in rodents before, but in our study, we showed that this effect can be observed even when animals’ eyes had adapted to complete darkness, as pupil dilation increases with the duration of cocaine intoxication [60]. In our study, we also used tracking points on the mouse head to detect specific movements of the animal under cocaine intoxication. Among the analyzed behaviors, particularly interesting were increased licking and jaw movements, which are also characteristic effects of a psychostimulant overdose in humans, colloquially known as gurning.

Drinking mentholatum (a strongly bitter tastant) was shown to elicit opposing effects on the mouse facial profile compared to drinking sucrose, making the face more convex, closing the eye, and rounding the snout [57]. We observed that quinine elicited a similar grimace – shortening the distance from eye to nose and the angle between nose, eye and lip. Collectively, these features differed most when compared to sucrose drinking.

It is uncertain whether changes in face profile observed in response to cocaine reflect pleasant sensations associated with drug effects, or if they are nonspecific manifestations of impaired circuitry controlling muscle movements. Cocaine exposure induced the biggest changes in most of the parameters we tracked. Some, such as shortening the distance between the eye and whiskers, were also elicited by sucrose. On the other hand, cocaine exposure reduced the angle between the eye and lip, which was also present in quinine drinking. Cocaine may trigger a mixture of sensations, which, combined with other movement-related effects (jaw movements, licking, increased locomotion), make it challenging to analyze.

To measure associations between cluster-specific neuronal dynamics and facial and locomotor behaviors, we calculated correlations for cocaine and sucrose/quinine sessions. Across all sessions, these correlations were cluster-specific, further demonstrating that distinct subpopulations of DRD1(+) and DRD2(+) neurons contribute differentially to sensorimotor processing. In cocaine sessions, although several neuronal clusters displayed statistically significant associations with facial and locomotor behaviors, the consistently modest correlation strengths suggest that cluster-specific activity of DRD1(+) and DRD2(+) neurons accounts only for a limited portion of the variance in behavioral measures, pointing to a more distributed and multifactorial neural control of cocaine-induced behavioral states. In sucrose/quinine sessions, we observed stimulus-dependent patterns of coupling between DRD1(+) and DRD2(+) neuronal cluster activity and facial behaviors. While DRD1(+) populations showed stronger and tightly time-locked associations during sucrose exposure, DRD2(+) populations exhibited more pronounced correlations during quinine, often with behavioral changes following neuronal activity. These findings indicate divergent temporal coordination and sensorimotor integration mechanisms between the two neuronal populations.

Similar high-resolution video recordings combined with large-scale neural recordings have been used in mice to link behavior to neural activity across cortical and deeper brain structures [26, 61–63]. Those results have shown that the activity of neurons can often be a result of widespread brain activation driven by locomotor activity [62], but also allowed to link orofacial movements to behaviorally tuned neuronal clusters across the cortex [63].

In the present study, we extend these approaches to the CeA, showing that at least among DRD1(+) and DRD2(+) populations, it is possible to distinguish neuronal clusters with different stimulus-specific correlations. While many previous studies attributed broad neuronal activation to locomotion or movements, our findings highlight more selective, subpopulation-level contributions to potentially differentiating appetitive and aversive stimuli. Determining the causal relationships of these clusters to behavioral responses will require targeted manipulations, such as cell-type-specific optogenetic stimulation or inhibition.

### Limitations of the study

While recording facial expressions, ear movements could not be tracked, as they were covered by the mounting bar used for head fixation. It was reported that mice experiencing gustatory stimuli tilt the ear forward, as opposed to tactile and painful stimuli, which tilt the ear backward [57]. Nevertheless, without the ability to properly track ear movements, we found that monitoring the frontal part of the mouse head was sufficient to detect certain changes in the mouse’s facial expression after exposure to rewards or bitter tastes.

In this study, we combined two different behavioral models to assess the activity of dopamine-sensitive neurons in the CeA. The two-photon imaging was performed using a protocol where mice were experiencing positive and aversive stimuli for the first time. Such an approach adds novelty to either rewarding or aversive sensations triggered by cocaine, sucrose, or quinine, making the data interpretation more challenging. As shown in Fig. 3F, there is a decrease in the frequency of calcium transients in DRD2 cells only after the first exposure to cocaine, not when it was repeated 1 or 2 times. There is no such effect on the activity of DRD1 cells. As sucrose and quinine were ingested only in one session, it should be noted that the novelty aspect of these new tastes will be significant.

As much as we are confident that 7.5% sucrose is appetitive, based on our previous research and vast literature data, we chose 0.1 mM quinine as it is mildly aversive [8, 64–67]. Using a higher quinine concentration would cause a stronger aversion, thus lowering the number of licks during the imaging session. However, future studies should involve titration of these substances to test multiple concentrations, as neutral stimuli for proper evaluation of the degree to which sucrose and quinine exert appetitive or aversive sensations.

The lack of a structured trial design of our experiment with cocaine made it difficult to temporally align behavioral events with neuronal signals in a statistically robust way. This is an inherent limitation of spontaneous behavior paradigms and could be addressed in future work through time-locked experimental designs.

While two-photon imaging enabled the identification of functional subpopulations of DRD1(+) and DRD2(+) neurons, technical limitations prevented reliable tracking of individual cells across days, primarily due to challenges in consistently imaging the same focal plane within the CeA. Although longitudinal single-cell tracking was not feasible, our population-level analysis captured dynamic recruitment and disengagement of CeM neurons across sessions in response to repeated drug exposure. Future work utilizing improved image registration methods or extended-depth imaging techniques (e.g., Bessel beams) could enable more precise longitudinal tracking and comparison of neuronal responses to both sucrose and cocaine within the same cells. Calcium imaging offers a powerful opportunity to monitor neuronal dynamics over time within the same animal. However, it is important to consider that repeated cocaine exposure may have influenced subsequent responses to sucrose and quinine, which should be taken into account when interpreting current findings.

Although the presence of both excitatory and inhibitory responses within each population supports a distributed coding scheme at the population level, the current experimental design does not allow us to determine whether individual neurons exhibit stable stimulus-specific tuning across different tastants or drugs. Longitudinal tracking of the same neurons across multiple stimulus sessions would be required to test whether single DRD1(+) or DRD2(+) neurons are consistently tuned to positive or negative valence, or whether valence information emerges primarily from flexible, distributed activity across the population. In our data DRD1(+) cells responded primarily to sucrose, whereas DRD2(+) cells responded primarily to quinine. Moreover, responses within these populations appeared stimulus-specific, with some cells increasing activity and others decreasing activity in response to the same stimulus. However, the presence of cells with opposite response directions (excitation and inhibition) within each recorded population suggests that, at the population level, valence may be encoded in a distributed manner. Given that the CeM is a major output of the amygdala and plays a key role in driving approach and avoidance behaviors in mice, the observed activity patterns could reflect opposing regulation of these behavioral responses. For example, in response to quinine, DRD2(+) cells might activate downstream neurons, promoting withdrawal while inhibiting those promoting approach. Similar functional antagonism has been demonstrated in the BLA using calcium imaging and holographic optogenetic stimulation [68]. In that study, stimulating appetitive ensembles increased quinine consumption, while stimulating aversive ensembles reduced sucrose intake. Whether similar effects arise from mutual inhibition between DRD1(+) and DRD2(+) cell ensembles in the CeM remains unknown.

Since our experiment did not track the same neurons across days, neuronal coding in the CeA during cocaine exposure could not be determined. Studies that monitor individual cells across sessions are needed to test whether specific neurons specialize in encoding the rewarding or aversive aspects of cocaine intoxication. Identifying such cells may provide targets for therapies, such as in alcohol addiction, as it was shown that optogenetic activation of prefrontal cortex projections to the periaqueductal gray (which convey aversive intoxication signals) reduces compulsive alcohol intake in mice [69].

It is also important to note that although we recorded activity from DRD1(+) and DRD2(+) neurons, this activity may not necessarily reflect direct dopaminergic modulation, as these cells could be activated via other neurotransmitter systems. Future studies using dopamine sensors (e.g., GRAB-DA) are needed to confirm the presence and timing of dopamine release within the CeA during stimulus exposure [70].

## Supplementary information

Supplemental information

## Data Availability

All data are available in the main text and the supplementary materials. Confocal images, behavioral data, and electrophysiological recordings https://doi.org/10.18150/TJDGZC Github repository with Matlab scripts for data analysis: https://github.com/BijochLukasz/Calcium-Imaging-Analysis-DRD1-vs-DRD2.
