# Supplementary material for: DRD1 and DRD2 dopamine-sensitive neurons in the central amygdala respond differently to rewarding and aversive stimuli
Source: Transl Psychiatry. 2026 Jul 3;16:494. doi: 10.1038/s41398-026-04228-7 (PMC13616905; doi:10.1038/s41398-026-04228-7)
Supplement: Supplementary file 1 [file 41398_2026_4228_MOESM1_ESM.pdf]

Supplementary Materials for

DRD1 and DRD2 dopamine-sensitive neurons in the central amygdala respond differently to rewarding and aversive stimuli

This file includes:

Figs. S1 to S9,
Table 1
Figure Legends
Supplementary Materials and Methods

Figures

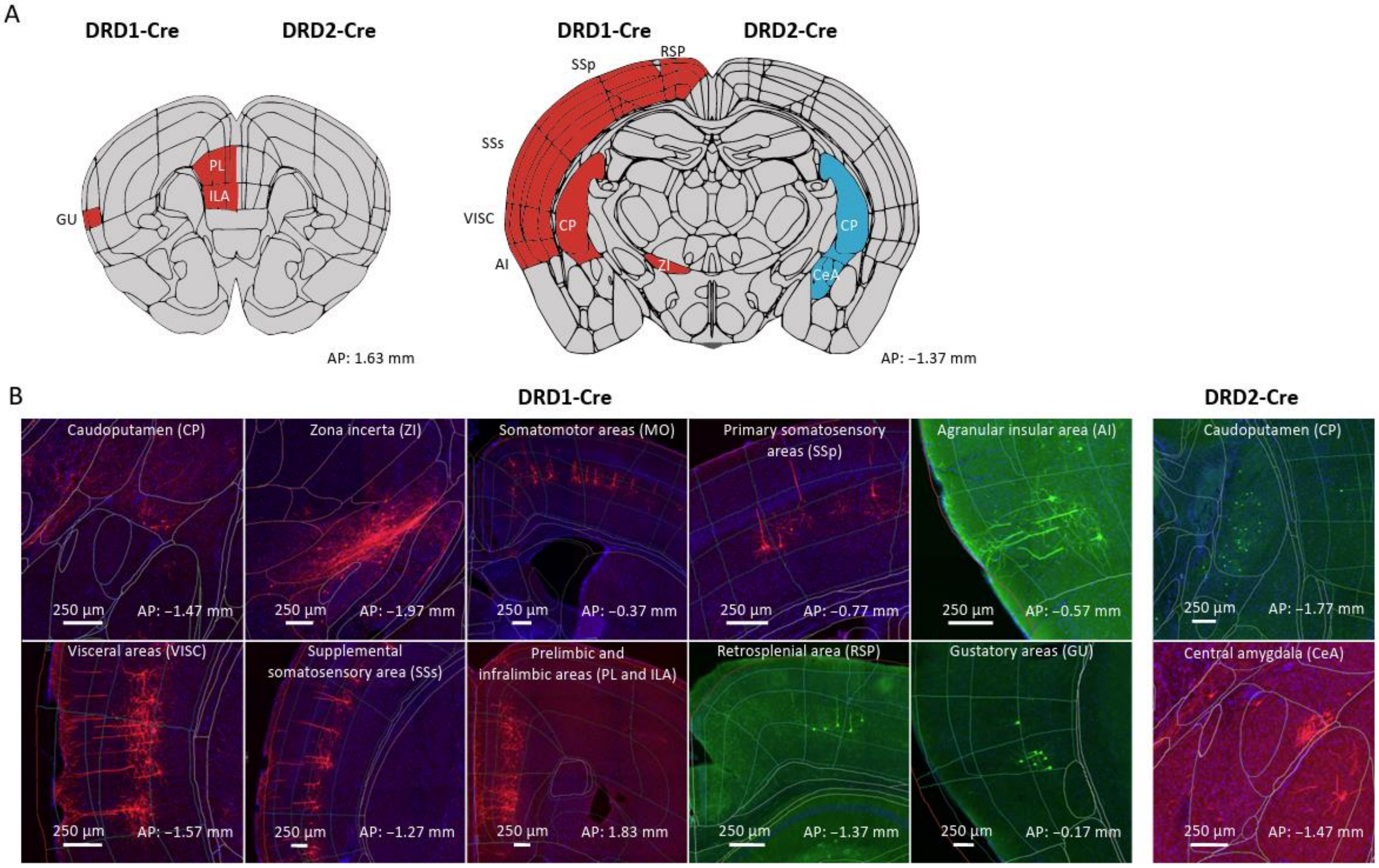

Fig. Supp. 1: Projections from dopamine-sensitive cells to the CeA (related to Fig. 1). A) Schematic representation of regions with DRD1(+) (red, left) and DRD2(+) (cyan, right) cells projecting to the CeA. AP in the lower-right corner indicates the anterior-posterior position (in mm) relative to bregma. **B**) Representative images of retrograde tracing of DRD1(+) and DRD2(+) neurons projecting to the CeA. AP in the lower-right corner indicates the anterior-posterior position (in mm) relative to bregma.

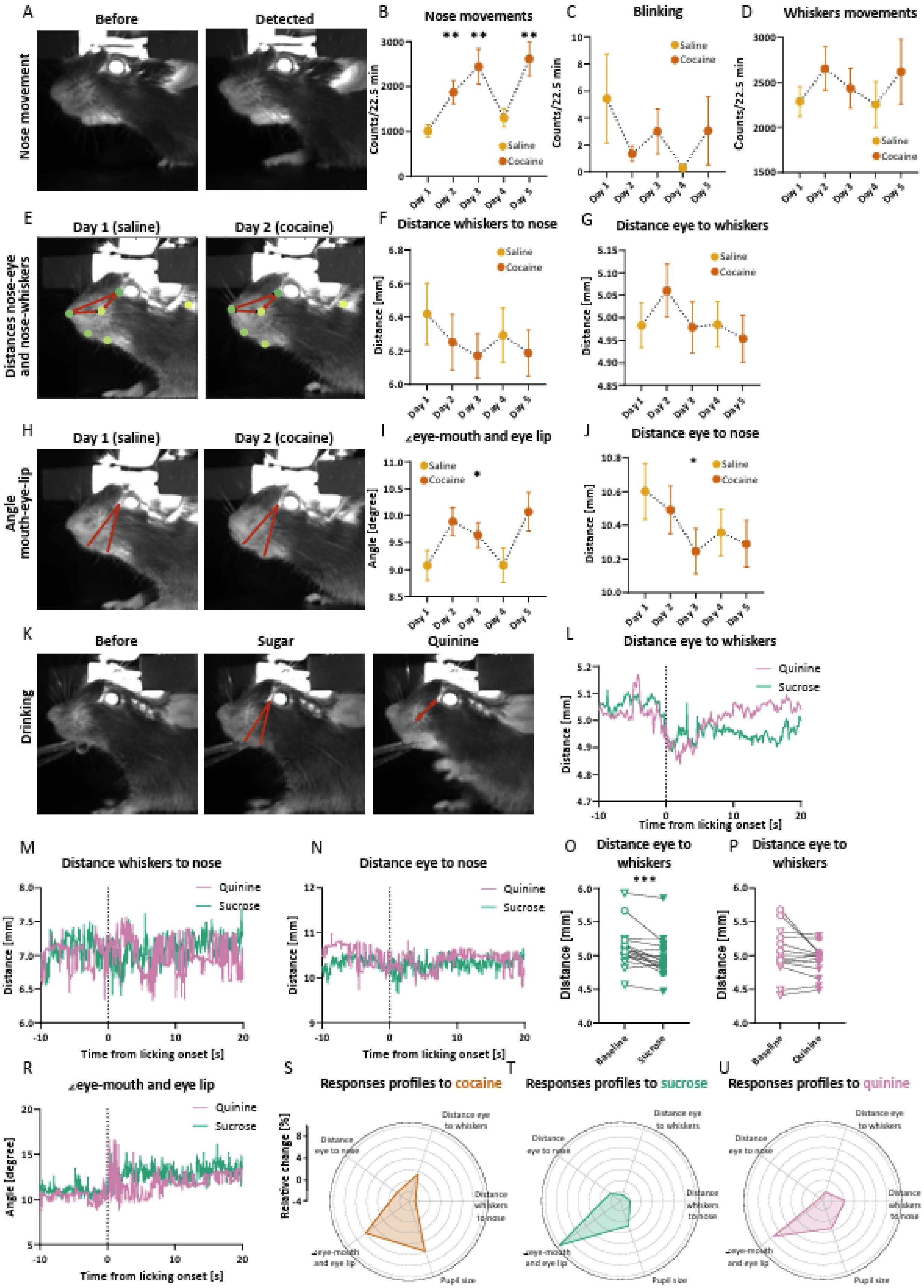
A
Before
Detected
Nose movement
B
Nose movements
Counts/22.5 min
Saline
Cocaine
C
Blinking
D
Whiskers movements
E
Day 1 (saline)
Day 2 (cocaine)
Distances nose-eye and nose-whiskers
F
Distance whiskers to nose
Distance [mm]
G
Distance eye to whiskers
H
Angle mouth-eye-lip
I
∡eye-mouth and eye lip
Angle [degree]
J
Distance eye to nose
K
Before
Sugar
Quinine
Drinking
L
Distance eye to whiskers
Quinine
Sucrose
Time from licking onset [s]
M
Distance whiskers to nose
N
Distance eye to nose
O
Distance eye to whiskers
Baseline
Sucrose
P
Distance eye to whiskers
Baseline
Quinine
R
∡eye-mouth and eye lip
S
Responses profiles to cocaine
Relative change [%]
Distance eye to whiskers
Distance eye to nose
Distance whiskers to nose
∡eye-mouth and eye lip
Pupil size
T
Responses profiles to sucrose
U
Responses profiles to quinine

Fig. Supp. 2: Facial expressions of mice after cocaine, sucrose, and quinine exposure (related to Fig. 2). A) Example frames from the video recordings before and during nose movement. **B**, **C**, **D**) Graphs representing events counted by SimBA: nose movements (**B**), blinking (**C**), whisker movements (**D**). Graphs represent averages for all mice ± SEM. **E**, **H**) Exemplary frames from a mouse exposed to saline (left) and cocaine (right) showing distances of tracked points (**E**) and angle between mouth and eye lip (**H**). Dots represent tracked points of the mouse face, and red arrows represent distances between the nose, whiskers, and eye. **F**, **G**, **I**, **J**) Graphs representing distances between whiskers and nose (**F**), distances between eye and whiskers (**G**), angles between mouth, lip, and eye (**I**), and distances between eye and nose during cocaine and saline sessions. Graphs represent averages for all mice ± SEM. **K**) Example frames of a mouse before and after a sugar and quinine droplet lick. Red arrows represent distances between eyes and whiskers. **L**-**N**, **R**) Graphs representing averaged distances between eye and whiskers (**L**), distances between whiskers and nose (**M**), distances between eye and nose (**N**), and angles between mouth, eye, and lip (**R**) 10 seconds before and 20 seconds after a lick of sucrose (green) and quinine (pink). **O**, **P**) Graphs representing averaged distances between the eye and whiskers before and after a lick of sucrose solution (**O**) or quinine solution (**P**). Each symbol represents the average for an individual mouse (triangles for males and circles for females). **S**) Response profiles to cocaine, sucrose, and quinine showing a percentage change of parameters from **F**-**R** and pupil size, relative to baseline (for sucrose and quinine) and saline (for cocaine).

N numbers in groups for **B**-**D**, **F**, **G**, **I**, and **J**: N = 17 (8♂, 9♀), where N = number of animals (number of males, number of females). For **P**-**S**: N sugar = 18 (10♂, 8♀) and N quinine = 14 (7♂, 7♀), where N = number of animals.

Statistical differences for **B, D, G, I** were measured with one-way ANOVA with repeated measures with Holm-Šídák's multiple comparisons test, with p-values for **B** day 1 vs. day 2 $p = 0.0042$, day 1 vs. day 3 $p = 0.0042$, day 1 vs. day 5 $p = 0.0042$, and for **I** day 1 vs. day 3 $p = 0.0483$. Statistical differences for **C, F, J** were measured with repeated-measures lognormal one-way ANOVA with Holm-Šídák's multiple comparisons test, with p-values for **J** day 1 vs. day 3 $p = 0.0470$. Statistical difference for **O** was measured with the Wilcoxon test, with p-value $p = 0.0008$. Statistical difference for **P** was measured with the paired t-test, with p-value $p = 0.0912$. Statistical differences are represented by stars, where *, **, and *** correspond respectively to $p < 0.05$, $p < 0.01$, and $p < 0.001$.

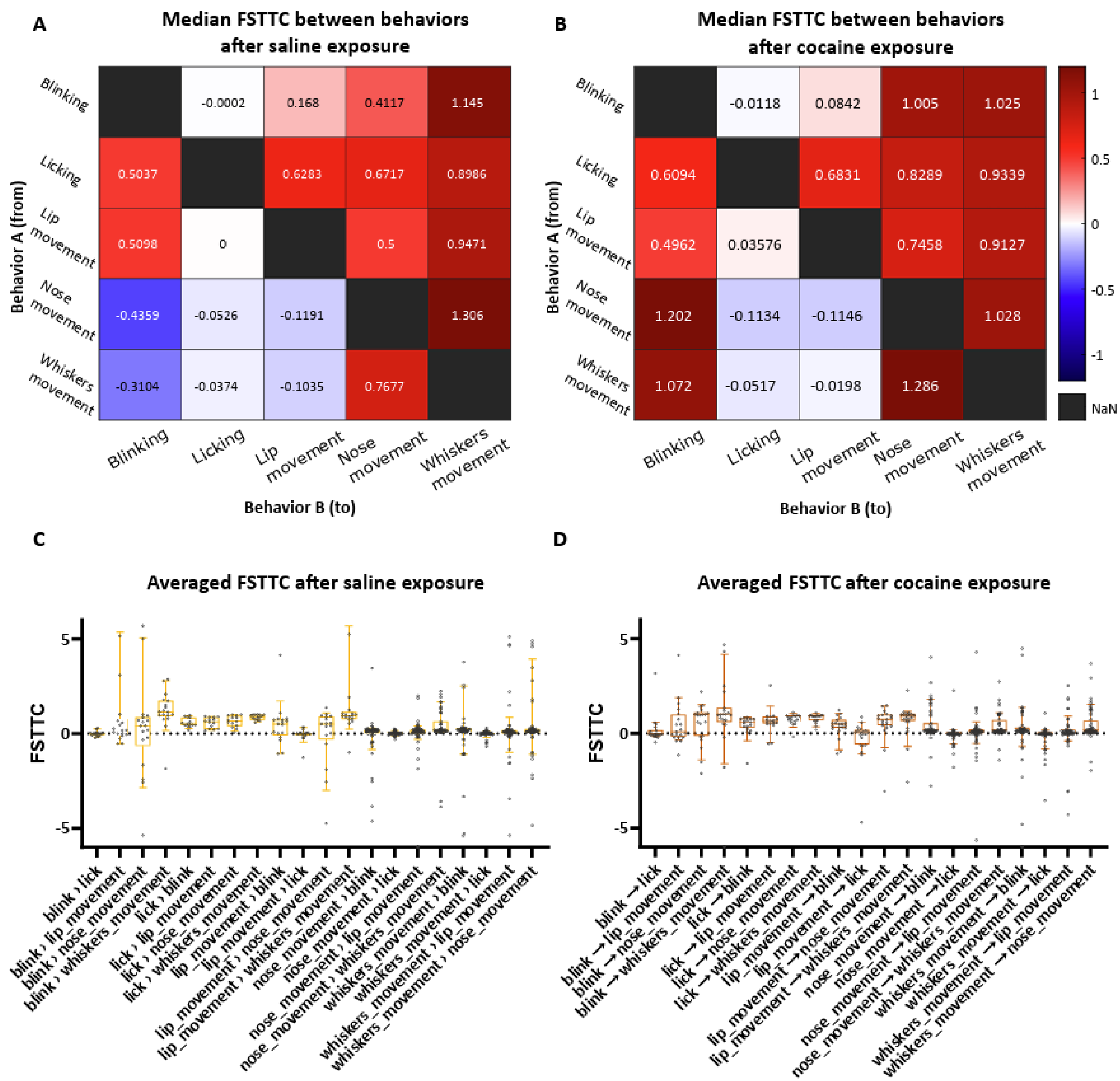

Fig. Supp. 3: Temporal organization of behavioral transitions measured using FSTTC. A) Heatmaps show average First-Second Time-Triggered Correlation (FSTTC) values between pairs of facial behaviors across animals treated with saline (left; averaged Day 1 and Day 4) or cocaine (right; averaged Day 2, 3, and 5). Higher FSTTC values indicate stronger temporal coupling between two behaviors (i.e., behavior A is reliably followed by behavior. **B)** Bar plots illustrate the distribution of FSTTC values across animals for each behavior pair. Graphs represent the median and 90-10 percentiles. Points represent the average values for mice. N numbers in groups: N = 17 (8♂, 9♀), where N = number of animals (number of males, number of females).

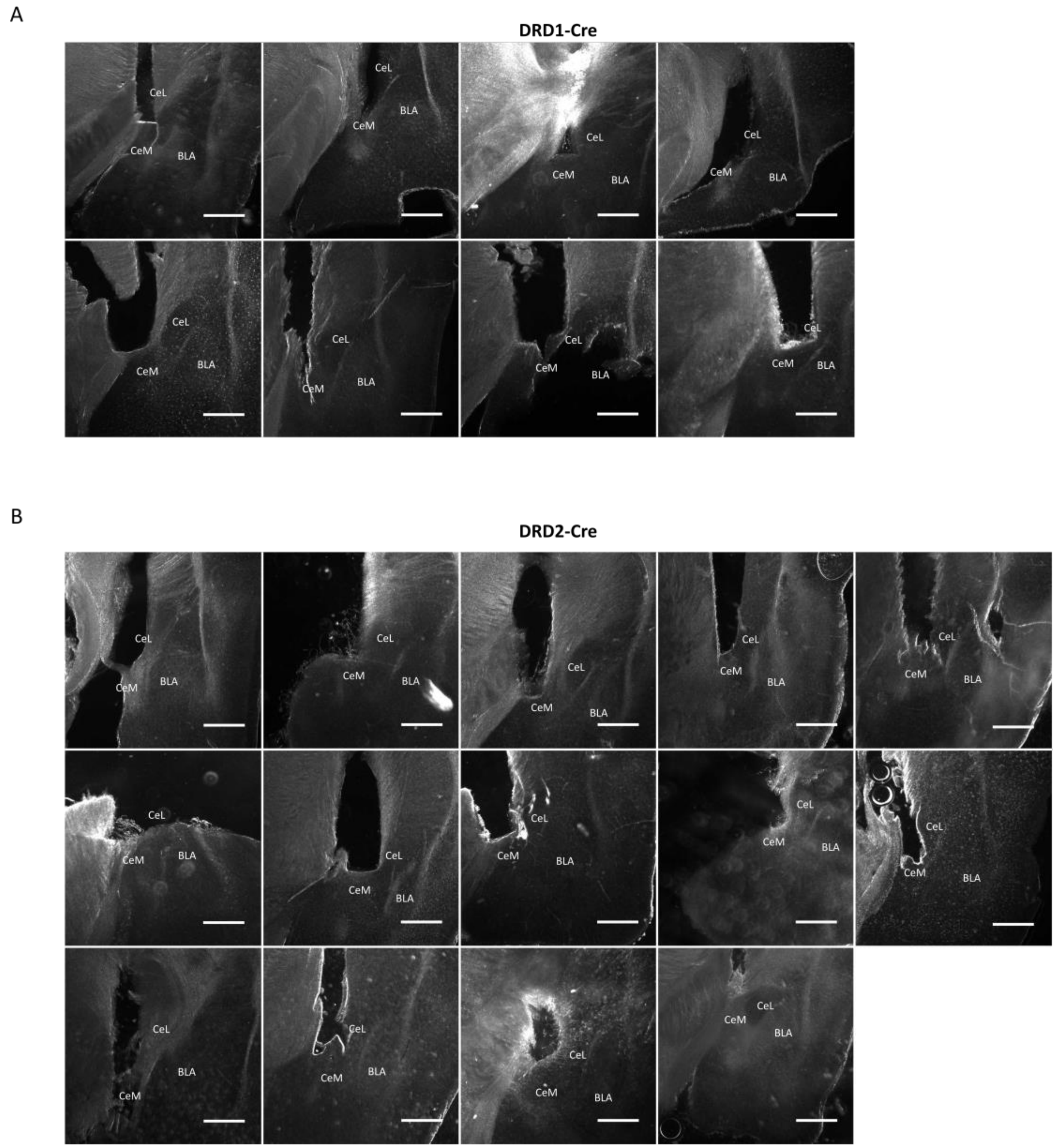

Fig. Supp. 4: Images of scars in the brain after GRIN lens insertion (related to Fig. 3). A, **B**) Images of scars in the brain after GRIN lens insertion from DRD1-Cre (**A**) and DRD2-Cre (**B**) mice. Scale bars 250 µm.

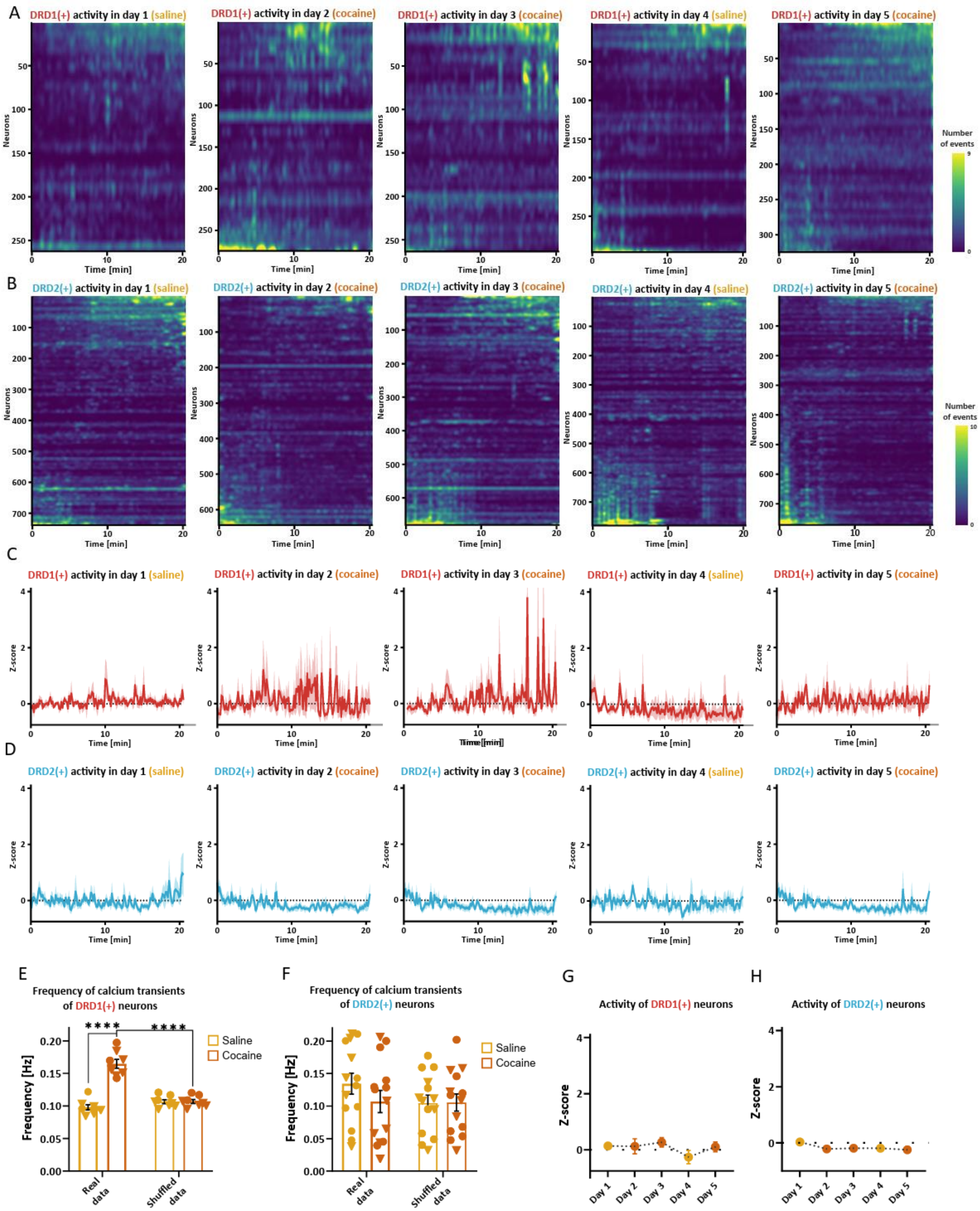

Fig. Supp. 5: Calcium activity of DRD1- and DRD2-positive neurons in the medial part of the central amygdala after cocaine exposure (related to Fig. 3). A, **B**) Heatmaps showing calcium activity as frequency of events of all recorded DRD1(+) (**A**) and DRD2(+) (**B**) neurons across the whole experiment. During each session, mice received either a saline or cocaine injection before imaging. Neurons were sorted based on the difference in their activity before and after 7 minutes of recording. **C**, **D**) Graphs showing averaged activity of neurons (calculated as z-score) from

DRD1(+) (**C**) and DRD2(+) (**D**) mice across the whole experiment. During each session, mice received either saline or a cocaine injection before imaging. **E**, **F**) Graphs showing the averaged frequency of neurons during saline days (day 1 and day 4) and cocaine days (day 2, day 3, and day 5) for real data and shuffled data. Graphs for DRD1(+) (**G**) and DRD2(+) (**H**) mice represent averages for all mice ± SEM. Each symbol represents the average of an individual mouse (triangles for males and circles for females). **G**, **H**) Graphs showing averaged activity of neurons (calculated as z-score) from DRD1(+) (**G**) and DRD2(+) (**H**) mice. Graphs represent averages for all mice ± SEM. Each symbol represents the average for an individual mouse (triangles for males and circles for females).
Number of groups for DRD1(+) N = 8 (5♂, 3♀) and for DRD2(+) N = 14 (6♂, 8♀), where N = number of animals (number of males, number of females).
Statistical differences for (**E**) and (**F**) were measured with two-way ANOVA with Holm-Šídák's multiple comparisons test, with p-value for **E** Real data Saline vs. Cocaine $p < 0.0001$ and Real data Cocaine vs. Shuffled data Cocaine $p < 0.0001$. Statistical differences for (**G**) and (**H**) were measured with paired one-way ANOVA.

A

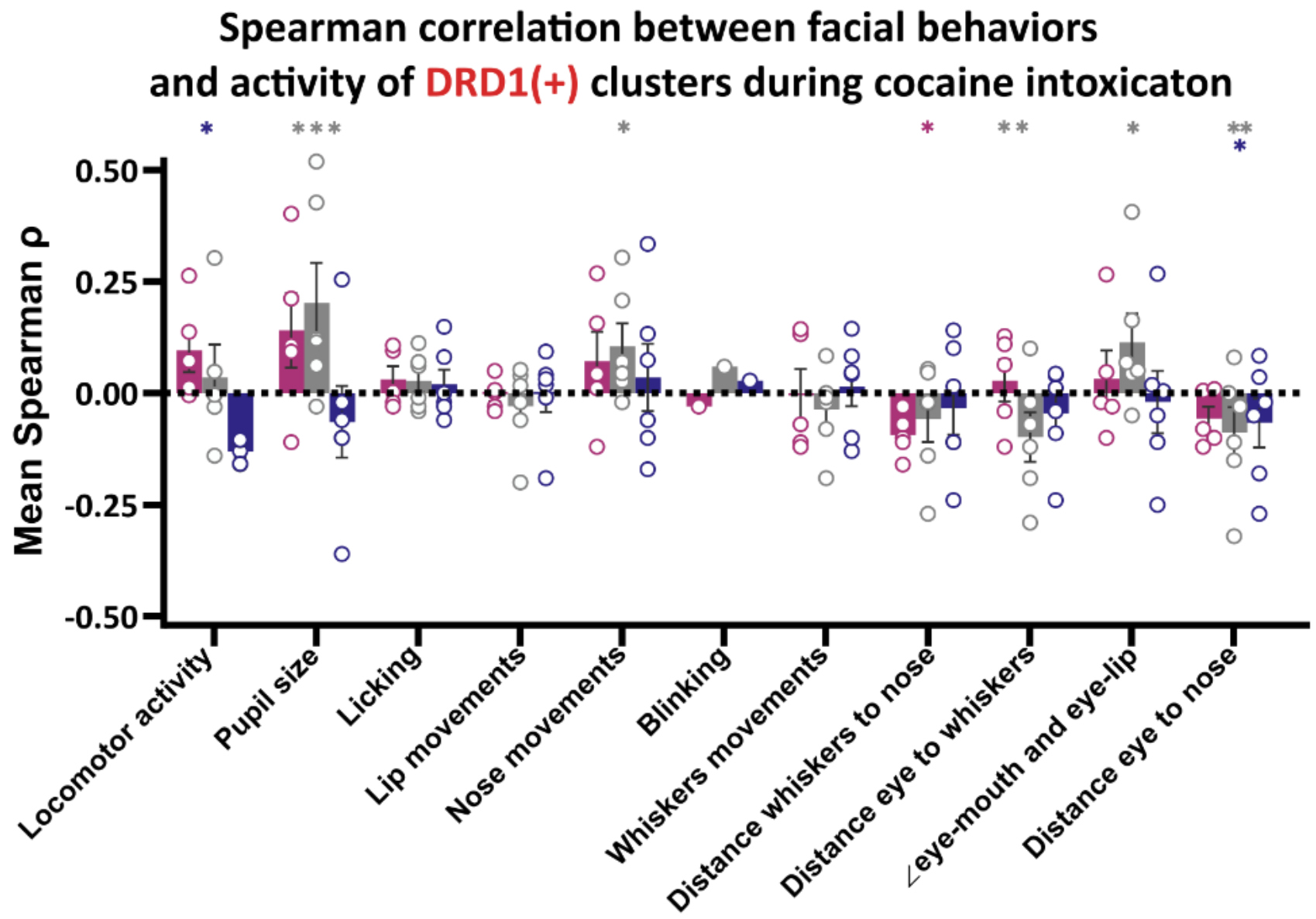

B

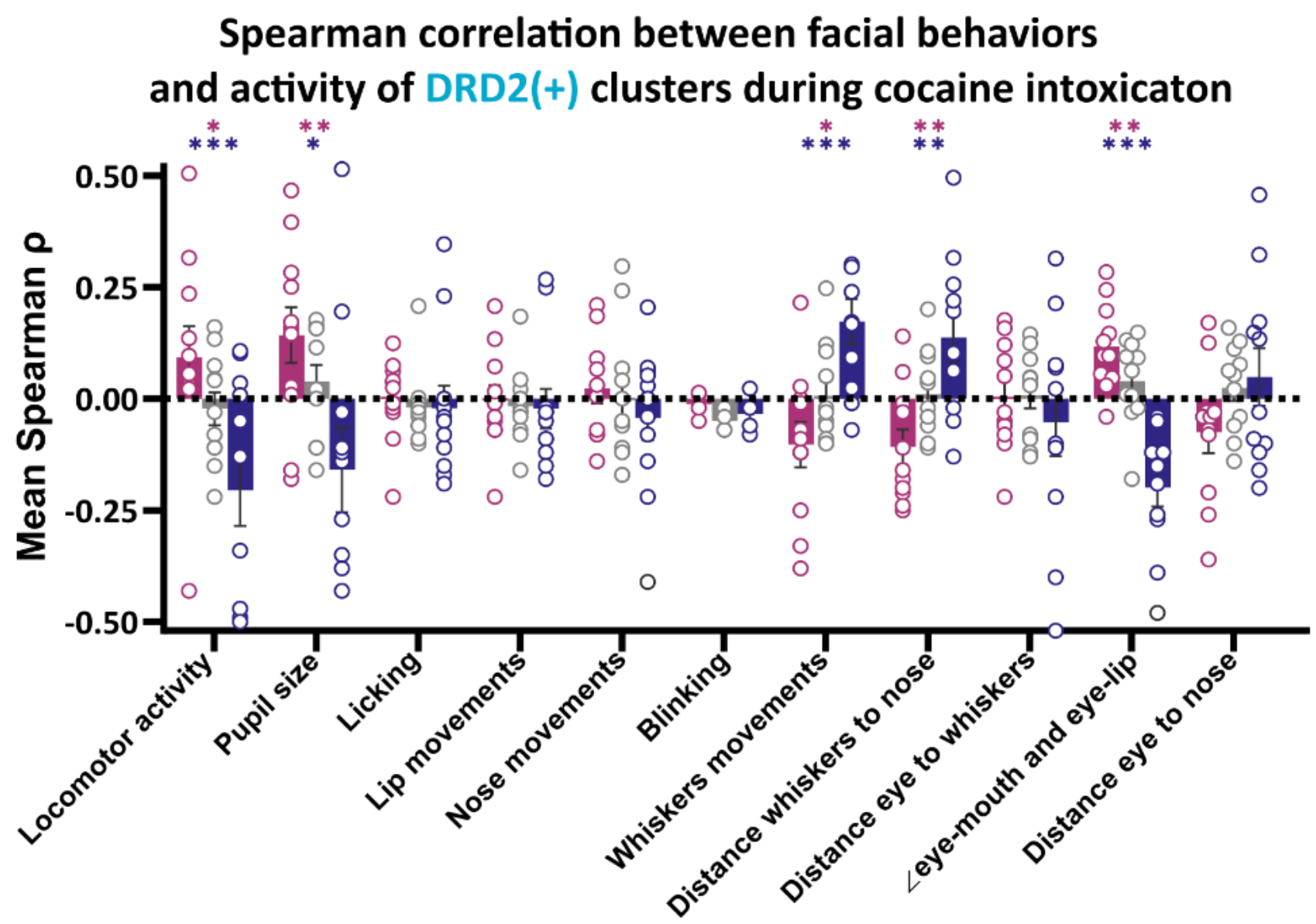

Fig. Supp. 6: Spearman correlations between activity of clusters of DRD1(+) and DRD2(+) neurons and behavioral parameters during cocaine exposure. A, **B**) Mean correlations between activity of DRD1(+) (**A)** and DRD2(+) (**B**) neuronal clusters and facial features of mice during first cocaine exposure. Correlations for different clusters are marked by different colors (magenta for Cluster I, grey for Cluster II and blue for Cluster III). Graphs represent averages for all mice ± SEM. Each symbol represents the correlation for an individual mouse. Statistical significance was assessed using a circular time-shift permutation test (2000 iterations), in which observed Spearman correlations between neuronal cluster activity and behavioral time series were compared to a null distribution generated from randomly shifted behavioral traces. To correct for multiple comparisons across clusters within each behavioral parameter, Holm–Bonferroni adjustment was applied. Statistical differences are represented by stars, where * correspond respectively to $p < 0.05$, ** to $p < 0.01$ and *** to $p < 0.001$. Statistics for different clusters are marked by different colors (magenta for Cluster I, grey for Cluster II and blue for Cluster III). Number of groups for DRD1(+) N = 6 (4♂, 2♀) and for DRD2(+) N = 11 (5♂, 6♀), where N = number of animals (number of males, number of females).

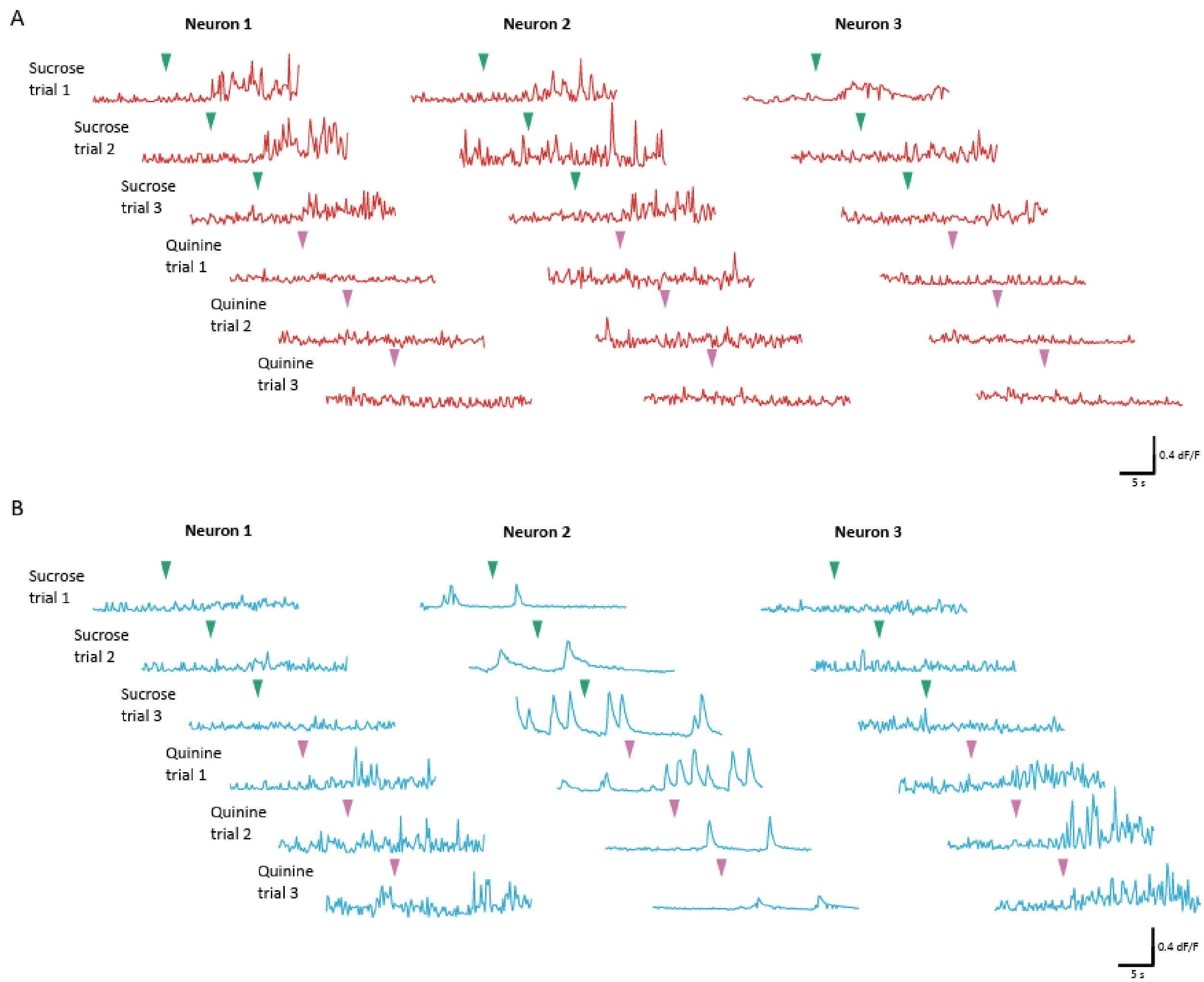

Fig. Supp. 7: Calcium activity of DRD1- and DRD2-positive neurons in the medial part of the central amygdala during sucrose and quinine exposure (related to Figure 4). A, **B**) Representative calcium traces of DRD1(+) (**A**, red) and DRD2(+) (**B**, blue) recorded during exposures of the animal to sucrose and quinine. Triangles represent the onset of lick of sucrose (green) and quinine (pink).

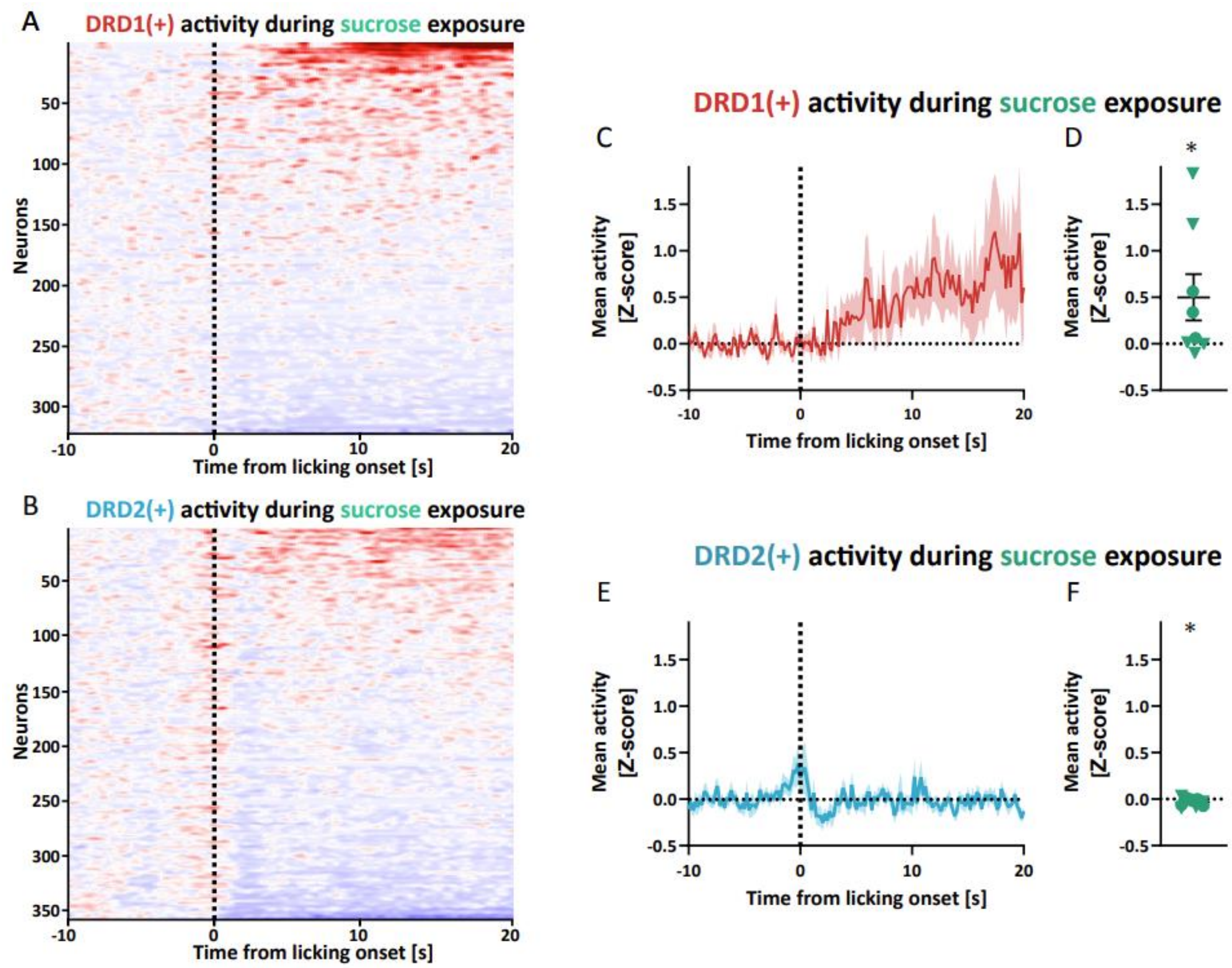

Fig. Supp. 8: Calcium activity of DRD1- and DRD2-positive neurons in the medial part of the central amygdala during sugar and quinine exposure (related to Fig. 4). A, **B**) Heatmaps showing calcium activity of all recorded DRD1(+) (**A**) and DRD2(+) (**B**) neurons averaged across all trials for 7.5% sucrose solution exposure. Z-scores for responses to sucrose were calculated for each neuron. Neurons were sorted based on the difference in their activity before and after the lick (the onset of the lick is marked with a dotted line). (**C**, **E**) Activity means of DRD1(+) (**C**) and DRD2(+) (**E**) neurons before and after a lick of sucrose solution, averaged for all mice. The onset of lick is represented as a vertical dotted line. (**D**, **F**) Graphs representing the averaged activity of DRD1(+) (**D**) and DRD2(+) (**F**) neurons after the lick of sucrose solution. Each dot represents the mean response of all neurons for each mouse (triangles for males and circles for females). Vertical lines represent averages for all mice ± SEM. **G**, **H**) Heatmaps showing calcium activity of all recorded DRD1(+) (**G**) and DRD2(+) (**H**) neurons averaged across all trials for 0.1mM quinine solution exposure. Z-scores for responses to quinine were calculated for each neuron. Neurons were sorted based on the difference in their activity before and after the lick (the onset of the lick is marked with a dotted line). (**I**, **K**) Activity means of DRD1(+) (**I**) and DRD2(+) (**K**) neurons before and after a lick of quinine solution, averaged for all mice. The onset of lick is represented as a vertical dotted line. (**J**, **L**) Graphs representing the averaged activity of DRD1(+) (**J**) and DRD2(+) (**L**) neurons after the lick of quinine solution. Each dot represents the mean response of all neurons for each mouse (triangles for males and circles for females). Vertical lines represent averages for all mice ± SEM.

N numbers in groups for sucrose exposure: DRD1(+) N = 8 (5♂, 3♀), for DRD2(+) N = 10 (5♂, 5♀). N numbers in groups for quinine exposure: DRD1(+) N = 6 (3♂, 3♀), for DRD2(+) N = 8 (4♂, 4♀), where N = number of animals. Statistical differences for (**D** and **L**) were measured with the Wilcoxon test (activity before the lick vs. activity after the lick), with p values for **D** $p = 0.0391$ and for **L** $p = 0.0391$. Statistical differences for (**F** and **J**) were measured with a paired t-test (activity before the lick vs. activity after the lick), with p values for **F** $p = 0.0444$ and for **J** $p = 0.9744$. Statistical differences are represented by stars, where * corresponds to $p < 0.05$.

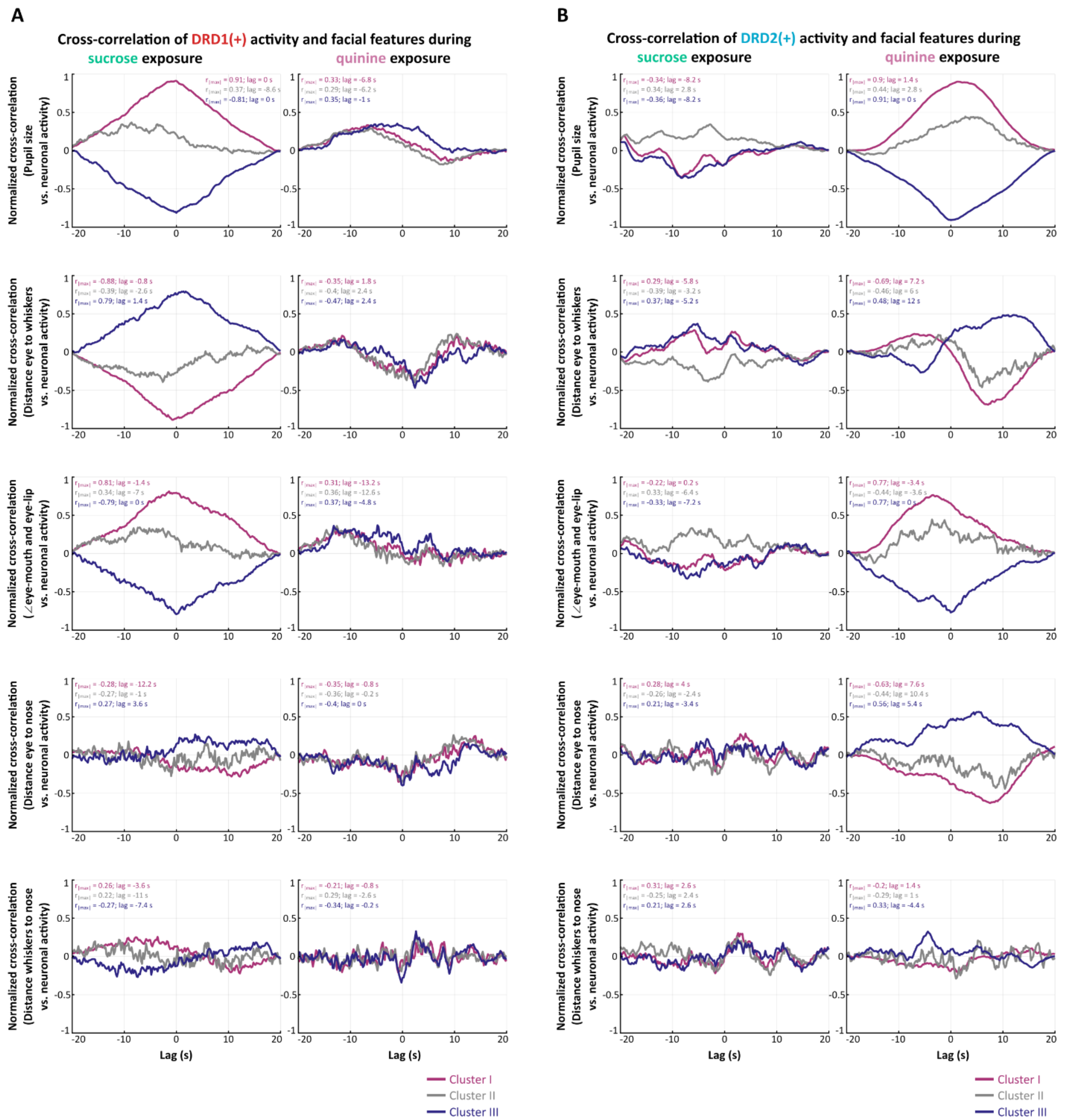

Fig. Supp. 9: Cross-correlations between activity of clusters of DRD1(+) and DRD2(+) neurons and behavioral parameters during sucrose and quinine exposure. A, B) Cross-correlations for activity of DRD1(+) (**A**) and DRD2(+) (**B**) neuronal clusters and facial features of mice during sucrose (left panels) and quinine (right panels) exposure. Graphs show normalized cross-correlations of each pair at different lags (in seconds). Behavioral changes preceded neuronal activity when the correlation was higher at lag < 0 or followed it when higher at lag > 0). Correlations for different clusters are marked by different colors (magenta for Cluster I, grey for Cluster II and blue for Cluster III). Maximal correlations for each cluster (r|max|) and their corresponding lags (lag) are in the top part of each graph (labeled with colors corresponding to clusters on the graph). Statistical significance was assessed using a permutation test, comparing the observed correlations between neuronal clusters and behavioral data to a null distribution generated from correlations with randomly shifted behavioral time series.

**Figure**	**Comparison**	**Test**	**F (DFn, DFd)/t,df/ W**	**p-value**	**ε (GG)**	**Normality (W, p)**	**Post-hoc Test**	**Post-hoc Results**
1B	Percentage of DRD1(+) and DRD2(+) cells in CeM	Paired t test	t=17.51, df=4	<0.0001	-	DRD1(+) (0.9995, 0.9590); DRD2(+) (0.9818, 0.7418)	-	-
1C	Percentage of DRD1(+) and DRD2(+) cells in CeL	Paired t test	t=1.238, df=4	0.2834	-	DRD1(+) (0.9293, 0.4861); DRD2(+) (0.8545, 0.2525)	-	-
2C	Locomotor activity	Repeated-measures lognormal one-way ANOVA	F (2.542, 47.65) = 52.36	<0.0001	0.6354	Day1 (0.7447, 0.0001); Day2 (0.8215, 0.0014); Day3 (0.8516, 0.0057); Day4 (0.7653, 0.0002); Day5 (0.9241, 0.1190)	Holm-Šídák's multiple comparisons test	Day 1 vs. Day 2: p < 0.0001; Day 1 vs. Day 3: p < 0.0001; Day 1 vs. Day 5: p < 0.0001 and Day 4 vs. Day 2: p < 0.0001; Day 4 vs. Day 3: p < 0.0001; Day 4 vs. Day 5: p < 0.0001
2F	Pupil size	One-way ANOVA with repeated measures	F (2.932, 43.24) = 6.812	0.0008	0.7330	Day1 (0.9592, 0.6777); Day2 (0.9726, 0.8790); Day3 (0.9586, 0.6367); Day4 (0.8920, 0.0600); Day5 (0.9027, 0.0753)	Holm-Šídák's multiple comparisons test	Day 1 vs. Day 2: p = 0.0326; Day 1 vs. Day 3: p = 0.0326; Day 1 vs. Day 5: p =0.0326 and Day 4 vs. Day 2: p = 0.0140; Day 4 vs. Day 3: p = 0.0140; Day 4 vs. Day 5: p = 0.0140
2I	Licking	Repeated-measures lognormal one-way ANOVA	F (2.908, 45.07) = 1.655	0.1912	0.7269	Day1 (0.6347, <0.0001); Day2 (0.7303, 0.0003); Day3 (0.7651, 0.0007); Day4 (0.6633, <0.0001); Day5 (0.6460, <0.0001)	Holm-Šídák's multiple comparisons test	Day 1 vs. Day 2: p = 0.0389; Day 1 vs. Day 3: p = 0.4786; Day 1 vs. Day 5: p =0.4786 and Day 4

								vs. Day 2: p = 0.0097; Day 4 vs. Day 3: p = 0.1683; Day 4 vs. Day 5: p = 0.1683
2J	Lip movement	Repeated-measures lognormal one-way ANOVA	F (2.894, 44.86) = 2.358	0.0863	0.7689	Day1 (0.8357, 0.0090); Day2 (0.7460, 0.0004); Day3 (0.8454, 0.0091); Day4 (0.8097, 0.0037); Day5 (0.8355, 0.0050)	Holm-Šídák's multiple comparisons test	Day 1 vs. Day 2: p = 0.0389; Day 1 vs. Day 3: p = 0.4786; Day 1 vs. Day 5: p =0.4786 and Day 4 vs. Day 2: p = 0.0097; Day 4 vs. Day 3: p = 0.1683; Day 4 vs. Day 5: p = 0.1683
2M	Pupil size	Wilcoxon test	95	0.0385	-	Baseline (0.8936, 0.0444); Response (0.8760, 0.0224)	-	-
2N	Pupil size	Paired t test	t=1.858, df=12	0.0879		Baseline (0.9023, 0.1439); Response (0.8885, 0.0929)	-	-
3D	Frequency of calcium transients of DRD1(+) neurons	Repeated-measures lognormal one-way ANOVA	F (1.914, 13.40) = 1.869	0.1931	0.4785	Day1 (0.9499, 0.7098); Day2 (0.7797, 0.0173); Day3 (0.951, 0.3911); Day4 (0.8874, 0.2215); Day5 (0.9338, 0.5517)	Holm-Šídák's multiple comparisons test	Day 1 vs. Day 2: p = 0.0485; Day 1 vs. Day 3: p = 0.0485; Day 1 vs. Day 5: p = 0.5416 and Day 4 vs. Day 2: p = 0.0647; Day 4 vs. Day 3: p = 0.9128; Day 4 vs. Day 5: p = 0.9128 and Day1 vs. Day4: p = 0.8769
3E	Frequency of calcium transients of DRD1(+)	Wilcoxon test	36	0.0078	-	Day 1(0.9499, 0.7098); Day2 (0.7797, 0.0173)	-	-

	neurons during Day1 and Day2							
3F	Frequency of calcium transients of DRD2(+) neurons	Repeated-measures lognormal one-way ANOVA	F (1.967, 24.58) = 1.770	0.197	0.4917	Day1 (0.9366, 0.3763); Day2 (0.8832, 0.0646); Day3 (0.9443, 0.5155); Day4 (0.8632, 0.0424); Day5(0.9115, 0.1658)	Holm-Šídák's multiple comparisons test	Day 1 vs. Day 2: p = 0.0251; Day 1 vs. Day 3: p = 0.4547; Day 1 vs. Day 5: p = 0.4095 and Day 4 vs. Day 2: p = 0.0647; Day 4 vs. Day 3: p = 0.9128; Day 4 vs. Day 5: p = 0.9128 and Day1 vs. Day4: p = 0.6388
3G	Frequency of calcium transients of DRD2(+) neurons during Day1 and Day2	Paired t test	t=2.491, df=13	0.0270	-	Day 1(0.9366, 0.3763); Day2 (0.8832, 0.0646)	-	-
3J	Mean responses of DRD1(+) clusters	Two-way ANOVA	F (2, 238) = 400.0	<0.0001	-	Baseline (0.9820, 0.7431); Response (0.9195, 0.4504)	Holm-Šídák's multiple comparisons test	Baseline vs. Response for Cluster I: p < 0.0001; for Cluster II p <0.0001; for Cluster III p = 0.0002
3K	Mean responses of DRD2(+) clusters	Two-way ANOVA	F (2, 639) = 425.4	<0.0001	-	Baseline (0.9240, 0.4667); Response (0.9092, 0.4153)	Holm-Šídák's multiple comparisons test	Baseline vs. Response for Cluster I: p < 0.0001; for Cluster II p = 0.0043; for Cluster III p < 0.0001
4D	Mean responses of DRD1(+) clusters	Two-way ANOVA	F (2, 174) = 64.66	<0.0001	0.5795	Baseline Sucrose (0.9945, 0.8578); Response Sucrose (0.9540, 0.8578);	Holm-Šídák's multiple compariso	(For Sucrose) Baseline Cluster I

						Baseline Quinine (0.7901, 0.0911); Response Quinine (0.8315, 0.1920)	ns test	vs. Response Cluster I: p < 0.0001; Baseline Cluster II vs. Response Cluster II: p = 0.0706; Baseline Cluster III vs. Response Cluster III: p < 0.0001; (For Quinine) Baseline Cluster I vs. Response Cluster I: p > 0.9999; Baseline Cluster II vs. Response Cluster II: p > 0.9999; Baseline Cluster III vs. Response Cluster III: p = 0.5298
4H	Mean responses of DRD2(+) clusters	Two-way ANOVA	F (2, 244) = 108.8	<0.0001	0.4554	Baseline Sucrose (0.9922, 0.8316); Response Sucrose (0.9192, 0.4495); Baseline Quinine (0.7816, 0.0713); Response Quinine (0.8871, 0.3455)	Holm-Šídák's multiple comparisons test	(For Sucrose) Baseline Cluster I vs. Response Cluster I: p = 0.8688; Baseline Cluster II vs. Response Cluster II: p = 0.5540; Baseline Cluster III vs. Response Cluster III: p = 0.8494; (For Quinine) Baseline Cluster I vs.

								Response Cluster I: p = 0.0140; Baseline Cluster II vs. Response Cluster II: p < 0.0001; Baseline Cluster III vs. Response Cluster III: p < 0.0001
S2B	Nose movements	One-way ANOVA with repeated measures	F (2.120, 32.87) = 5.950	0.0055	0.5301	Day1 (0.9718, 0.8675); Day2 (0.9383, 0.2985); Day3 (0.9241, 0.1732); Day4 (0.9420, 0.3748); Day5 (0.9536, 0.4836)	Holm-Šídák's multiple comparisons test	Day 1 vs. Day 2: p = 0.0042; Day 1 vs. Day 3: p = 0.0042; Day 1 vs. Day 5: p = 0.0042 and Day 4 vs. Day 2: p = 0.1677; Day 4 vs. Day 3: p = 0.0641; Day 4 vs. Day 5: p = 0.0163
S2C	Blinking	Repeated-measures lognormal one-way ANOVA	F (3.007, 46.60) = 0.6761	0.5714	0.5715	Day1 (0.4624, <0.0001); Day2 (0.6447, <0.0001); Day3 (0.5025, <0.0001); Day4 (0.4964, <0.0001); Day5 (0.3087, <0.0001)	Holm-Šídák's multiple comparisons test	Day 1 vs. Day 2: p = 0.9911; Day 1 vs. Day 3: p = 0.9454; Day 1 vs. Day 5: p = 0.9950 and Day 4 vs. Day 2: p = 0.5336; Day 4 vs. Day 3: p = 0.4809; Day 4 vs. Day 5: p = 0.3481
S2D	Whiskers movements	One-way ANOVA with repeated measures	F (2.069, 32.07) = 0.6853	0.5159	0.5172	Day1 (0.9098, 0.1154); Day2 (0.9723, 0.8570); Day3 (0.9700, 0.8178); Day4 (0.9084, 0.1095); Day5 (0.9632, 0.6644)	Holm-Šídák's multiple comparisons test	Day 1 vs. Day 2: p = 0.4461; Day 1 vs. Day 3: p = 0.4831; Day 1 vs. Day 5: p =

								0.4831 and Day 4 vs. Day 2: p = 0.5802; Day 4 vs. Day 3: p = 0.7265; Day 4 vs. Day 5: p = 0.6607
S2F	Distance whiskers to nose	Repeated-measures lognormal one-way ANOVA	F (2.946, 48.61) = 2.410	0.0793	0.7364	Day1 (0.7856, 0.0018); Day2 (0.6784, <0.0001); Day3 (0.8212, 0.0031); Day4 (0.7079, <0.0001); Day5 (0.7995, 0.0008)	Holm-Šídák's multiple comparisons test	Day 1 vs. Day 2: p = 0.2936; Day 1 vs. Day 3: p = 0.1346; Day 1 vs. Day 5: p = 0.2235 and Day 4 vs. Day 2: p = 0.2972; Day 4 vs. Day 3: p = 0.2235; Day 4 vs. Day 5: p = 0.2235
S2G	Distance eye to whiskers	One-way ANOVA with repeated measures	F (2.875, 47.44) = 0.8650	0.4619	0.7188	Day1 (0.9829, 0.9826); Day2 (0.9730, 0.8513); Day3 (0.9330, 0.2190); Day4 (0.9689, 0.7777); Day5 (0.9588, 0.5206)	Holm-Šídák's multiple comparisons test	Day 1 vs. Day 2: p = 0.2717; Day 1 vs. Day 3: p = 0.9716; Day 1 vs. Day 5: p > 0.9999 and Day 4 vs. Day 2: p = 0.2288; Day 4 vs. Day 3: p = 0.9999; Day 4 vs. Day 5: p = 0.9992
S2I	Angle eye-mouth and eye-lip	One-way ANOVA with repeated measures	F (2.467, 40.71) = 3.286	0.0382	0.6168	Day1 (0.9323, 0.2654); Day2 (0.9733, 0.8566); Day3 (0.9552, 0.5131); Day4 (0.9235, 0.1492); Day5 (0.9722, 0.8009)	Holm-Šídák's multiple comparisons test	Day 1 vs. Day 2: p = 0.0559; Day 1 vs. Day 3: p = 0.0483; Day 1 vs. Day 5: p = 0.0559and Day 4 vs. Day 2: p = 0.0087; Day 4 vs.

								Day 3: p = 0.0005; Day 4 vs. Day 5: p = 0.1653
S2J	Distance eye to nose	Repeated-measures lognormal one-way ANOVA	F (2.417, 39.88) = 3.802	0.0240	0.6043	Day1 (0.8991, 0.0777); Day2 (0.8893, 0.0375); Day3 (0.8131, 0.0023); Day4 (0.7723, 0.0006); Day5 (0.8062, 0.0011)	Holm-Šídák's multiple comparisons test	Day 1 vs. Day 2: p = 0.7822; Day 1 vs. Day 3: p = 0.0470; Day 1 vs. Day 5: p = 0.0611 and Day 4 vs. Day 2: p = 0.1025; Day 4 vs. Day 3: p = 0.0346; Day 4 vs. Day 5: p = 0.9119
S2O	Distance eye to whiskers	Wilcoxon test	-131	0.0008	-	Baseline (0.8693, 0.0214); Response (0.8688, 0.0210)	-	-
S2P	Distance eye to whiskers	Paired t test	t=1.824, df=13	0.0912	-	Baseline (0.9577, 0.6851); Response (0.9257, 0.2651)	-	-
S3C, D	Averaged FSSTC after saline and cocaine exposure	Two-way ANOVA with repeated measures	F (19, 244) = 1.664	0.0430	-	Saline (0.9795, 0.9268); Cocaine (0.9381, 0.2203)	-	-
S5E	Real data vs. shuffled data frequency of DRD1(+) cells	Two-way ANOVA with repeated measures	F (1, 14) = 106.9	< 0.0001	-	Real data: Saline days (0.9544, 0.7557), Cocaine days (0.9585, 0.7960); Shuffled data: Saline days (0.8918, 0.2432), Cocaine days (0.9345, 0.5582)	Holm-Šídák's multiple comparisons test	Saline days vs. Cocaine days for shuffled p = 0.9468 and real data p < 0.0001; Shuffled data vs. real data for Saline; p = 0.1762, for Cocaine p < 0.0001
S5F	Real data vs. shuffled data frequency of DRD2(+) cells	Two-way ANOVA with repeated measures	F (1, 26) = 2.114	0.1579	-	Real data: Saline days (0.9495, 0.5536), Cocaine days (0.9627, 0.7666); Shuffled	Holm-Šídák's multiple comparisons test	Saline days vs. Cocaine days for shuffled p

						data: Saline days (0.9256, 0.2645), Cocaine days (0.9169, 0.1987)		= 0.1109 and real data p = 0.9485; Shuffled data vs. real data for Saline; p = 0.2966, for Cocaine p = 0.9365
S5G	Comparison of z-scores of DRD1(+) neurons	One-way ANOVA with repeated measures	F (2.660, 15.96) = 1.391	0.2810	0.6651	Day1 (0.9847, 0.4598); Day2 (0.9515, 0.5846); Day3 (0.9072, 0.1678); Day4 (0.9072, 0.1678); Day5(0.9189, 0.2117)	Holm-Šídák's multiple comparisons test	Day 1 vs. Day 2: p = 0.9847; Day 1 vs. Day 3: p = 0.2380; Day 1 vs. Day 5: p = 0.9847 and Day 4 vs. Day 2: p = 0.2421; Day 4 vs. Day 3: p = 0.0641; Day 4 vs. Day 5: p = 0.2882
S5H	Comparison of z-scores of DRD2(+) neurons	One-way ANOVA with repeated measures	F (2.445, 30.56) = 1.545	0.2271	0.6112	Day1 (0.9431, 0.4598); Day2 (0.9515, 0.5846); Day3 (0.9072, 0.1678); Day4 (0.9072, 0.1678); Day5(0.9189, 0.2117)	Holm-Šídák's multiple comparisons test	Day 1 vs. Day 2: p = 0.2483; Day 1 vs. Day 3: p = 0.3302; Day 1 vs. Day 5: p = 0.3959 and Day 4 vs. Day 2: p = 0.9683; Day 4 vs. Day 3: p = 0.9932; Day 4 vs. Day 5: p = 0.9337
**Figure**	**Comparison**	**Test**	**Correlation for p 0.05 (r-value, p-value)**					
S6A	Correlations of activity of DRD1(+) neuronal clusters and facial features during cocaine exposure	Spearman correlation, Circular shift permutation test	Locomotor activity vs. Cluster III: (0.013, -0.1312), Pupil size vs. Cluster II: (<0.001, 0.2016), Nose movements vs. Cluster II: (0.0175, 0.105), Distance whiskers to nose vs. Cluster I: (0.029, -0.094), Distance eye to whiskers vs. Cluster II: (0.0004, -0.0593), Angle eye-mouth and eye-lip vs. Cluster II: (0.0105, 0.1135), Distance eye to nose vs. Cluster II: (0.003, -0.089), Distance eye to nose vs. Cluster III: (0.0029, -0.0686)					

S6B	Correlations of activity of DRD2(+) neuronal clusters and facial features during cocaine exposure	Spearman correlation, Circular shift permutation test	Locomotor activity vs. Cluster I: (0.04, 0.0938), Locomotor activity vs. Cluster III: (<0.0001, 0.1426), Pupil size vs. Cluster I: (0.0075, 0.2016), Pupil size vs. Cluster III: (0.023, -0.1596), Whiskers movements vs. Cluster I: (0.011, -0.1027), Whiskers movements vs. Cluster III: (<0.0001, 0.1724), Distance whiskers to nose vs. Cluster I: (0.003, -0.1086), Distance whiskers to nose vs. Cluster III: (0.003, 0.1379), Angle eye-mouth and eye-lip vs. Cluster I: (0.002, 0.1172), Angle eye-mouth and eye-lip vs. Cluster III: (<0.0001, -0.2002)					
**Figure**	**Comparison**	**Test**	**F (DFn, DFd)/t,df/ W**	**p-value**	**ε (GG)**	**Normality (W, p)**	**Post-hoc Test**	**Post-hoc Results**
S8D	Baseline vs. response of DRD1(+) activity during sucrose exposure	Wilcoxon test	26	0.0391	-	Baseline (0.8019, 0.030); Response (0.8224, 0.0495)	-	-
S8D’	Response of shuffled data vs. actual response of DRD1(+) activity during sucrose exposure	Wilcoxon test	28	0.0273	-	Response of shuffled data (0.9339, 0.5522); Actual response (0.8224, 0.0495)	-	-
S8F	DRD2(+) activity during sucrose exposure	Paired t test	t=2.335, df=9	0.0444	-	Baseline (0.9613, 0.8005); Response (0.9514, 0.6845)	-	-
S8F’	Response of shuffled data vs. actual response of DRD2(+) activity during sucrose exposure	Paired t test	t=3.463, df=9	0.0071	-	Baseline (0.9192, 0.3501); Response (0.9514, 0.6845)	-	-
S8J	DRD1(+) activity during quinine exposure	Paired t test	t=0.03372, df=5	0.9744	-	Baseline (0.8695, 0.2243); Response (0.9619, 0.8346)	-	-
S8J’	Response of shuffled data vs. actual response of DRD1(+) activity during quinine exposure	Paired t test	t=1.729, df=5	0.1443	-	Response of shuffled data (0.9620, 0.8347); Actual response (0.9619, 0.8346)	-	-
S8L	DRD2(+) activity during quinine exposure	Wilcoxon test	30	0.0391	-	Baseline (0.7448, 0.0072); Response (0.8308, 0.0606)	-	-

S8L’	Response of shuffled data vs. actual response of DRD2(+) activity during quinine exposure	Paired t test	t=2.143, df=7	0.0693	-	Baseline (0.9210, 0.4377); Response (0.8308, 0.0606)	-	-
**Figure**	**Comparison**	**Test**	**Correlation for $\lvert r\rvert < 0.5$ (r-value, p-value)**					
S9A	Correlations of activity of DRD1(+) neuronal clusters and facial features during sucrose and quinine exposure	Cross-correlation, Circular shift permutation test	For sucrose: Angle eye-mouth and eye-lip vs. Cluster I: (0.8124, 0.0395), Angle eye-mouth and eye-lip vs. Cluster III: (-0.7942, 0.008), Pupil size vs Cluster I: (0.9114, 0.0055), Pupil size vs Cluster III: (-0.813, 0.0055), Distance eye to whiskers vs. Cluster I: (-0.8846, 0.0405), Distance eye to whiskers vs. Cluster III: (0.7919, 0.113)					
S9B	Correlations of activity of DRD2(+) neuronal clusters and facial features during sucrose and quinine exposure	Cross-correlation, Circular shift permutation test	For quinine: Angle eye-mouth and eye-lip vs. Cluster I: (0.7655, 0.0533), Angle eye-mouth and eye-lip vs. Cluster III: (-0.7703, 0.022), Pupil size vs Cluster I: (0.9032, 0.0667), Pupil size vs Cluster III: (-0.9081, 0.0427), Distance eye to whiskers vs. Cluster I: (-0.686, 0.4333), Distance eye to nose vs. Cluster I: (-0.6274, 0.37), Distance eye to nose vs. Cluster III: (0.5648, 0.2427)					

Table 1: Statistical Analysis Summary. This table presents the figure, comparison conducted, statistical test applied, and corresponding test statistics: F (DFn, DFd) for ANOVA, t (df) for t-tests, or W for Wilcoxon tests. It also includes p-values, ε (Greenhouse-Geisser correction) for ANOVA, normality test results (Shapiro-Wilk W, p), post-hoc tests used, and post-hoc results.

Materials and Methods

Immunolabeling and confocal imaging

Mice were perfused with cold PBS, followed by 4% PFA dissolved in PBS. Their brains were removed and kept in 4% PFA for 24 hours at 4°C and later in 4°C PBS. Brains were sliced on a vibratome (Leica VT 1200 S) into 100 µm (for immunolabeling) or 250 µm (for GRIN lens location validation) slices.

For immunolabeling, brain slices (AP –1.17 to –1.67 mm from bregma) were permeabilized at room temperature in 0.25% Triton X-100 in PBS and then incubated for three hours in a blocking solution (3% BSA and 0.1% Triton X-100 in PBS). Slices were then incubated for 24 hours at 0°C in 0.1% Triton X-100 and 0.1% BSA in PBS containing anti-Cre antibodies (1:3000 [1], produced in Division of Molecular Genetics DKFZ) and with secondary antibody ThermoFisher A32733 that had been previously centrifuged at 20000g for 10 minutes.

Brain slices were placed on coverslips with a mounting medium (Fluoromont-G-DAPI) and imaged using Zeiss LSM 780 confocal microscope with 10x (dry, NA = 0.3) and 40X (oil, NA = 1.4) objectives. Diode lasers (405 nm and 561 nm) and an argon laser (488 nm) were used for

fluorescence excitation. Signals were collected at the spectra 340-350 nm, 510-530 nm, and 575-590 nm. Images were later analyzed with a Cell-Counter plugin in FIJI software.

Retrograde labeling of the dopamine-sensitive neurons projecting to the CeA

DRD2-Cre or DRD1-Cre mice were anesthetized with 5% isoflurane and injected i.p. with painkillers and anti-inflammatory drugs (butorphanol 3 mg/kg and tolfenamic acid 3 mg/kg). 50 nl of adeno-associated viruses (pAAV-hSyn-DIO-EGFP at titer 1,5 x 10*8; Addgene 50457-AAVrg or retrograde hSyn-DIO-mCherry at titer 3,75 x 10*8; Addgene 50459-AAVrg) were stereotactically injected into the CeA (coordinates from bregma: ML +2.2, AP -1.3, DV 4.6 mm) at a rate of 10 nl/min. After surgery, the welfare of the mice was monitored over the next 5 days. If needed, mice received painkillers and antibiotics (butorphanol 3 mg/kg, enrofloxacin 5 mg/kg, and tolfenamic acid 3 mg/kg). Three weeks post-surgery mice were perfused with cold PBS followed by 4% PFA dissolved in PBS. Their brains were removed and they were kept in 4% PFA for 24 hours in the 4°C and later in 4°C PBS. Brains were sliced on a vibratome (Leica VT 1200 S) into 50 μm slices. Brain slices were permeabilized at room temperature in 0.25% Triton X-100 in PBS and then incubated for 3 h in a blocking solution (3% BSA and 0.1% Triton X-100 in PBS). Slices were then incubated for 24 hours at 4°C in 0.1% Triton X-100 and 0.1% BSA in PBS containing a mix of antibodies against endogenous fluorophores: anti-GFP (Abcam ab13970, 1:1500) and anti-RFP (ThermoFisher M11217, 1:1500). Later, brain slices were incubated for 3 h at room temperature with a mix of secondary antibodies (ThermoFisher A-11039 or Abcam ab150158, 1:400) that had been previously centrifuged at 20000g for 10 minutes. Brain slices were mounted on coverslips and imaged with the fluorescent microscope (Olympus VS110). Images were then registered on Allen Brain Atlas[2] with *Aligning Big Brains* & *Atlases* software (ABBA, BioImaging & Optics Platform at EPFL, https://biop.github.io/ijp-imagetoatlas/)[3].

Two-photon microscope

Calcium imaging of CeM neurons was conducted using a custom-built two-photon microscope, based on the project described by Yao P et al. [4]. The microscope was equipped with a resonant scanning mirror operating at an 8 kHz scanning rate. The signal was collected at 512x512 pixels at 30 Hz, with each frame averaged 3 times. For a two-photon excitation, a water-cooled Halite 920 laser (Fluence) was used. The incorporated laser dispersion management allowed for optimal pulse compression to acquire the best signal-to-noise ratio. We used the 20x Nikon plan-fluor objective with NA = 0.5, which matched the NA of the GRIN lens. Data collection was performed using ScanImage software (mbf BIOSCIENCE).

1 Kellendonk, C. *et al.* Inducible site-specific recombination in the brain. *Journal of molecular biology* **285**, 175-182, doi:10.1006/jmbi.1998.2307 (1999).
2 Allen Institute for Brain Science (2011). Allen Reference Atlas – Mouse Brain [Adult Mouse]. Available from https://atlas.brain-map.org/.
3 Chiaruttini, N. *et al. ABBA, a novel tool for whole-brain mapping, reveals brain-wide differences in immediate early genes induction following learning* (2024).
4 Yao, P., Liu, R., Broggini, T., Thunemann, M. & Kleinfeld, D. Construction and use of an adaptive optics two-photon microscope with direct wavefront sensing. *Nature protocols* **18**, 3732-3766, doi:10.1038/s41596-023-00893-w (2023).
